# Mining of Novkitasetaline, a New Sulfur-Containing Antimalarial β-Carboline Alkaloid, from *Streptomyces* sp. PRh3 by Functional Ribosome Engineering Directed Heterologous Expression

**DOI:** 10.3390/microorganisms13122871

**Published:** 2025-12-18

**Authors:** Xingyu Chen, Xiaohui He, Yanmin Wang, Yangping Feng, Zihan Wang, Chunhui Song, Xinyu Yu, Yunchang Xie

**Affiliations:** 1 Key Laboratory of Biodiversity Conservation and Bioresource Utilization of Jiangxi Province, College of Life Sciences, Jiangxi Normal University, Nanchang 330022, China; 15279862930@163.com (X.C.); wym0826333@163.com (Y.W.);; 2 National Health Commission Key Laboratory of Parasitic Disease Control and Prevention, Jiangsu Provincial Key Laboratory on Parasite and Vector Control Technology, Jiangsu Institute of Parasitic Diseases, Wuxi 214064, China; hexiaohui@jipd.com; 3 Center for Global Health, School of Public Health, Nanjing Medical University, Nanjing 211166, China

**Keywords:** β-carboline alkaloid, ribosome engineering, heterologous expression, *Streptomyces*, antimalarial activity

## Abstract

The endophytic *Streptomyces* sp. PRh3 (PRh3), isolated from Dongxiang wild rice (DXWR), exhibited impaired biosynthetic capacity in the laboratory. To address this defect, rifampicin-based ribosome engineering was first applied to PRh3 to generate PRh3-r55, which acquired a characteristic H473Y rifampicin-resistant mutation in *rpoB* to activate the production of two β-carboline alkaloids JBIR-133 and JBIR-134. Then the biosynthetic gene cluster (BGC) *ksl* was introduced into PRh3-r55 for heterologous expression, generating PRh3-r55K. This combined approach achieved a synergistic effect, enabling the strain to produce not only the expected JBIR-133, JBIR-134, and kitasetaline, but also a novel sulfur-containing molecule, novkitasetaline. Structural elucidation identified novkitasetaline as a unique tryptamine-substituted kitasetaline derivative at the C-3 position of its pyridine ring. Notably, this structural modification conferred significant antimalarial activity to novkitasetaline, rendering it active against drug-sensitive *Plasmodium falciparum* 3D7 (IC_50_ = 32.65 ± 2.93 μM) and three other drug-resistant *P. falciparum* strains: K13^C580Y^, Dd2, and HB3 (IC_50_ = 45.98 ± 4.17~59.67 ± 3.15 μM), primarily by disrupting late-stage parasite development. These efforts not only identified a promising antimalarial lead compound but also demonstrated that combining ribosome engineering with heterologous expression is an effective strategy for discovering bioactive natural products from *Streptomyces*.

## 1. Introduction

*Streptomyces* can produce numerous secondary metabolites (SMs) [1,2,3]. These bioactive natural products (NPs) serve as feasible lead compounds in innovative drug development [2,3,4]. However, full-scale mining of the valuable SMs encoded in the genomes of various wild-type (WT) *Streptomyces* strains remains a challenge [5]. Firstly, the obvious difference between the laboratory environment and bacterial natural habitats could hamper SM production by repressing the expression of their biosynthetic gene clusters (BGCs) in routine strain cultivation [5,6]. Directly activating these silent BGCs requires precise genomic information or mature genetic manipulation tools, which are often unavailable for WT strains [7,8]. Thus, to overcome the aforementioned barriers, the relatively simple and versatile ribosome engineering has been employed to stimulate the production of *Streptomyces* SMs recently [9,10,11]. This strategy, derived from the research regarding bacterial “stringent response”, positively regulates their secondary metabolism, can efficiently generate desirable SM-producing mutants simply by treating *Streptomyces* strains with rifampicin [9,12]. During the “stringent response”, strains can produce abundant signal molecule ppGpp to bind the ppGpp-sensitive domain in the β-subunit of RNA polymerase (RNAP) [9,12]. This binding could modulate RNAP activity by enhancing the gene expression involved in SM biosynthesis [9,12,13]. Coincidentally, the ppGpp-sensitive domain is located close to the rifampicin binding domain in RNAP β-subunit [9,12]. *Streptomyces* strains treated with rifampicin can acquire resistant mutations in *rpoB*, which will encode the corresponding mutant β-subunit to form relevant resistant mutant RNAP in generated mutant strains [9,12]. Subsequent binding of rifampicin to the mutant RNAP, similarly to the abovementioned ppGpp binding to its sensitive domain in RNAP, could also activate the expression of biosynthetic genes to produce bioactive SMs in *Streptomyces* strain [9,12,13].

As a straightforward strategy, ribosome engineering is independent of the target strains’ mature genetic condition [12]. Thus, this reliable strategy is quite suitable for dealing with difficult *Streptomyces* strains without complete genome information and established genetic manipulation systems [9,11]. In this study, *Streptomyces* sp. PRh3 (PRh3) is a new endophytic strain isolated from Dongxiang wild rice (DXWR), a wild crop with the highest latitude distribution and excellent environmental adaptability [14]. In view of PRh3’s distinct habitat, the comprehensive fermentation coupled with metabolite profiling was performed. However, these laboratory assays failed to detect any meaningful SM from this strain or to acquire its high-quality whole-genome sequence. Due to this abnormal situation, a conventional ribosome engineering treatment (rifampicin in concentration gradient) was adopted to improve the secondary metabolism in PRh3 and generated an expected resistant mutant strain PRh3-r55, which acquired an H473Y rifampicin-resistant mutation in *rpoB* and was activated to produce a small amount of two β-carboline alkaloids, JBIR-133 and JBIR-134.

Previous research has revealed that *Kitasatospora setae* NBRC 14216^T^, harboring BGC *ksl*, can produce JBIR-133, JBIR-134, and the additional β-carboline alkaloid, kitasetaline [15,16,17]. Nevertheless, identification of *ksl*-homologous BGC in PRh3 was blocked due to a lack of genome sequence, which also, in return, hampered the subsequent discovery of β-carboline alkaloids from PRh3. Therefore, given the consistency of producing JBIR-133 and JBIR-134 in the above two strains, we adopted a substituted heterologous expression by overexpressing *ksl* in PRh3-r55 and generated the derivative mutant strain PRh3-r55K. This attempt not only effectively enhanced the production of JBIR-133 and JBIR-134, but also achieved a synergistic effect between the heterologous expression of *ksl* and the optimized intracellular secondary metabolic environment of the host strain, which enabled PRh3-r55K to produce kitasetaline and novkitasetaline. Novkitasetaline is a derivative of kitasetaline, where the carboxyl group at C-3 of the pyridine ring forms an amide bond with tryptamine. This structural modification significantly enhanced the molecules’ antimalarial activity. Novkitasetaline exhibited potency against *Plasmodium falciparum* 3D7 with an IC_50_ value of 32.65 ± 2.93 μM. In contrast, its precursor, kitasetaline, exhibited no obvious antimalarial bioactivity. Notably, novkitasetaline also exhibited activity against the drug-resistant *P. falciparum* strains with IC_50_ of 45.98 ± 4.17 μM (K13^C580Y^), 51.88 ± 4.76 μM (Dd2), and 59.67 ± 3.15 μM (HB3). These consistent activities against both drug-sensitive and drug-resistant parasites revealed that novkitasetaline possessed a relatively broad antimalarial spectrum. Furthermore, novkitasetaline exerted peak antimalarial activity against late-stage parasites (specifically the trophozoite-to-schizont transition phase) by either arresting parasite growth or impairing merozoite invasion. In conclusion, this study successfully activated the biosynthetic potential of β-carboline alkaloids in PRh3 by combining ribosome engineering with the heterologous expression of *ksl*. This combinatorial strategy not only obtained a new sulfur-containing antimalarial β-carboline alkaloid, novkitasetaline, but also provided new insight into mining of bioactive NPs from *Streptomyces*.

## 2. Materials and Methods

### 2.1. Strain and Culture Condition

PRh3 was isolated from DXWR, Jiangxi province, China (28°14′ N, 116°30′ E). This strain has been deposited into the Guangdong Microbial Culture Collection Center (GDMCC) with the number GDMCC 65483. This strain and its mutants were cultivated on a modified-ISP4 (M-ISP4) plate [18]. For each fermentation, 50 μL of spore suspension (10^7^ spores) of PRh3 and its derived mutant strains was inoculated into 50 mL liquid M-ISP4 in the 250 mL flasks, then incubated at 28 °C and 200 rpm for 6 days. The 16S ribosomal RNA gene sequence of PRh3 was uploaded to the GenBank with accession Number PV855736.1.

### 2.2. Ribosome Engineering of PRh3

Spore suspensions (10^6^–10^7^ spores) of PRh3 were spread onto an M-ISP4 plate containing different concentrations (5–100 μg/mL) of rifampicin and cultivated at 28 °C for persistent observation. Then the minimal inhibition concentration (MIC) of rifampicin against PRh3 was determined at 22 μg/mL. The following treatments of PRh3 were carried out by applying rifampicin at concentrations of 1 × MIC-5 × MIC on the M-ISP4 plates to obtain resistant mutant strains. Genetic characterization of the *rpoB* mutation in the final selected rifampicin-resistant mutant strain PRh3-r55 was performed by PCR using four pairs of primers (Table S1). The amplified oligonucleotides were cloned onto the *pEASY*^®^-T&B simple cloning Vector (Transgen biotech; Beijing, China) and sequenced to verify the mutations. The sequence of *rpoB* was also uploaded to the GenBank with accession Number PV870206.1.

### 2.3. Heterologous Expression of ksl in PRh3 and PRh3-r55

To effectively activate the expression of *ksl*, the necessary corresponding vector pJN44 was designed and constructed. This vector was derived from pSET152. Firstly, the pSET152 was digested by EcoRI and XbaI to yield a related linear vector. Then, two sequence-complementary oligonucleotides containing cohesive ends of EcoRI and XbaI were annealed to form the corresponding double-stranded fragment by touchdown polymerase chain reaction (PCR) (Table S2). This double-stranded product also contained the promoter SP44, ribosome binding site (RBS) SR40, and two additional restriction enzyme recognition sites: NdeI and BamHI [19]. Finally, the double-stranded DNA was ligated into previously prepared linear pSET152 to generate the pJN44.

The pJN44 was digested by NdeI and Xba1 to yield the linear vector. Then the full-length (3389 bp) *ksl* was amplified from the genomic DNA of *Kitasatospora setae* NBRC 14216^T^ by using one pair of primers (Table S3). Subsequently, the *ksl* fragment was ligated into the linear pJN44 to generate the pJN44-*ksl* by using the “LightNing^®^ DNA Assembly Mix Plus” (BestEnzymes; Lianyungang, China).

The pJN44-*ksl* was subsequently transformed into the PRh3 and PRh3-r55 by *Escherichia coli* ET12567/pUZ8002 mediated conjugation [20]. Target exconjugants were subsequently selected on M-ISP4 plates supplied with 50 μg/mL apramycin to confirm their antibiotic resistance. Then, single colonies were patched onto M-ISP4 plates containing 50 μg/mL apramycin, and the correct PRh3-r55K was further verified by PCR and sequencing (Table S3).

### 2.4. Strain Fermentation, Product Isolation, and Identification

To isolate JBIR-133 and JBIR-134 from PRh3-r55, a 2-step fermentation process was adopted. First, the spores of PRh3-r55 were inoculated into 50 mL liquid M-ISP4 in the 250 mL flask and cultured at 28 °C and 200 rpm for 36 h. Then, these seed cultures were transferred into 200 mL liquid M-ISP4 in the 1 L flask and cultured at 28 °C and 200 rpm for an additional 7 days. At last, about 5 L cultures were harvested in this way. All the culture broth was centrifuged and divided into the supernatant and the mycelium cake. The supernatant was extracted with equal ethyl acetate three times and evaporated to dryness. The mycelium was extracted with acetone and evaporated to dryness too. These two extracts were dissolved and mixed with silica gel (100–200 mesh) for normal phase silica gel column chromatography, which was eluted by CH_2_Cl_2_/MeOH mixture at 100/0, 98/2, 96/4, 95/5, 93/7, 90/10, 80/20, and 50/50 to yield 8 fractions (Afr1–Afr8). The Afr3 was then subjected to the following gel permeation chromatography to yield an additional 52 fractions (Bfr1-Bfr52). The Bfr11-Bfr14 were mixed and purified by semi-preparative HPLC with a YMC-Pack ODS-A column (250 mm × 10 mm, 5 μm) to give the resulting 4.1 mg JBIR-133 and 3.3 mg JBIR-134.

To isolate JBIR-133, JBIR-134, kitasetaline, and novkitasetaline from PRh3-r55k, another 2-step fermentation process was adopted. The spores of PRh3-r55k were also inoculated into 50 mL liquid M-ISP4 in the 250 mL flask and cultured at 28 °C and 200 rpm for 36 h. Then, the seed culture was transferred into 250 mL liquid M-ISP4 in the 1 L flask and cultured at 28 °C and 200 rpm for an additional 6 days. At last, about 20 L of cultures were harvested in this way. All the culture broth was centrifuged and divided into the supernatant and the mycelium cake. The supernatant was extracted with equal ethyl acetate three times and evaporated to dryness. The mycelium was extracted with acetone and evaporated to dryness too. These two extracts were dissolved and mixed with silica gel (100–200 mesh) for normal phase silica gel column chromatography, which was eluted by CH_2_Cl_2_/MeOH mixture at 100/0, 98/2, 96/4, 95/5, 93/7, 90/10, 80/20, and 50/50 to yield 8 fractions (Afr1–Afr8). The Afr3–Afr4 was then subjected to the following gel permeation chromatography to yield an additional 102 fractions (Bfr1–Bfr102). The Bfr16–Bfr22 were mixed and purified by semi-preparative HPLC with a YMC-Pack ODS-A column (250 mm × 10 mm, 5 μm) to give the resulting 15.1 mg JBIR-133 and 9.2 mg JBIR-134, respectively. The Bfr47–Bfr48 was mixed and purified by the same semi-preparative HPLC to yield 4.2 mg kitasetaline and 3.5 mg novkitasetaline, respectively.

Analytic HPLC of β-carboline alkaloids was performed at 280 nm with an Agilent 1260 Infinity series instrument (Santa Clara, CA, USA) equipped with a diode array detector (DAD) and an Agilent ZORBAX SB-C18 column (250 mm × 4.6 mm, 5 μm). The solvent system consists of solvents A (water with 0.1% trifluoroacetic acid) and B (acetonitrile with 0.1% trifluoroacetic acid). The elution process was run under the following conditions: 5% B to 50% B (linear gradient, 0–15 min), 50% B to 80% B (linear gradient, 15–20 min), 80% B to 100% B (linear gradient, 20–21 min), 100% B (isocratic elution, 21–24 min), 100% B to 5% B (linear gradient, 24–25 min), and 5% B (isocratic elution, 25–30 min). The flow rate is set at 1 mL/min. Optical rotation was measured on an Anton Paar MCP 5300 polarimeter (Graz, Austria) at 25 °C and a wavelength of 589 nm. High-resolution electrospray ionization mass spectrometry (HRESIMS) data were acquired on a ZenoTOF 7600 system (AB SCIEX, Framingham, MA, USA), using a syringe pump operated at 0.4 mL/min. The solvent system consists of solvents A (water) and B (acetonitrile). The elution process was run under the following conditions: 10% B (isocratic elution, 0–0.6 min), 10% B to 95% B (linear gradient, 0.6–1 min), 95% B (isocratic elution, 1–3.5 min), 95% B to 10% B (linear gradient, 3.5–3.6 min), 10% B (isocratic elution, 3.6–5 min). Full scan data were acquired in the positive ionization mode using a spray voltage of 5.5 kV. Nuclear magnetic resonance (NMR) spectra were acquired at 298 K on a Bruker Avance IIIHD 600M, a Bruker Avance NEO 400M, and a Bruker Avance IIIHD 700M (Bruker, Bremen, Germany) spectrometer equipped with a 5 mm TCI CryoProbe (Bruker, Germany), using standard Bruker pulse sequences and phase cycling. NMR data were analyzed using the MestReNova v14.0 software (Mestrelab Research, Santiago de Compostela, Spain).

### 2.5. Antimalarial Activity Assay

#### 2.5.1. Ethics Statements

This study was carried out in accordance with the institutional guidelines for the care and use of biosamples in Jiangsu Institute of Parasitic Diseases (JIPD). The experimental design was reviewed and approved by the Research Ethics Committee of JIPD (JIPD-2022-005).

#### 2.5.2. Parasite Culturing

Plasmodium falciparum strains—including the wild-type (3D7), artemisinin (ART)-resistant strain (K13^C580Y^), chloroquine (CQ)-resistant strain (Dd2), and pyrimethamine (PYR)-resistant strain (HB3)—were cultured in RPMI 1640 medium supplemented with 0.5% (*w*/*v*) Albumax I, 50 mg/L hypoxanthine, 25 mM NaHCO_3_, 25 mM HEPES, and 10 mg/L gentamycin, and contained 2% hematocrit of O^+^ erythrocytes (supplied by Wuxi Blood Center, Wuxi, Jiangsu, China). Parasite cultures were maintained at 37 °C under an atmosphere of 5% CO_2_, 5% O_2_, and 90% N_2_, as previously reported [21]. Parasites were routinely synchronized via two consecutive treatments with 5% sorbitol, and late-stage parasites were purified using 40/70% Percoll density gradient centrifugation. Parasitemia was quantified microscopically using Giemsa-stained thin blood smears.

#### 2.5.3. Asexual Growth Inhibition Assay

A 3-day SYBR Green Growth inhibition assay was conducted based on the 3-day SYBR Green I method for the determination of the IC_50_ value. Specifically, 100 μL of ring-stage parasites obtained from sorbitol synchronization were incubated in a 96-well plate at 0.5% parasitemia and 2% hematocrit. Cultures were incubated with 2-fold serially diluted compounds ranging from 6.25 to 500 μM in a total volume of 200 μL for an additional 72 h under normal conditions. Then, 100 μL lysis buffer (30 mM Tris-HCl, 7.5 mM EDTA, 0.12% saponin, and 0.12% Triton X-100) containing 5 × SYBR green I was added to each well. Parasite cultures were lysed and stained in the dark for an additional 2 h at RT, and parasite-related fluorescence was monitored via a microplate reader with excitation and emission wavelengths at 485 nm and 530 nm, respectively. Each plate contained a vehicle-treated control and a negative control (erythrocyte only at 2% hematocrit) for background subtraction, and the growth inhibition assay was performed by two independent experiments with technical triplicates each time. IC_50_ was calculated by fitting the data with a nonlinear regression curve using GraphPad Prism 8.0.

#### 2.5.4. Stage-Specific Antimalarial Assay

To assess the stage-specific antimalarial activity, parasites were subjected to double synchronization using 5% sorbitol at 40 h intervals. Following this, a 40/70% Percoll treatment was implemented, and the remaining parasites were cultured with fresh erythrocytes for 3 h to promote merozoite invasion. Highly synchronized parasites at ring (0–3 hpi), trophozoite (24 ± 2 hpi), and schizont (32 ± 2 hpi) were incubated with varied concentrations of the compound for 16 h in the 96-well plates at a starting parasitemia of 1%. The survival rate for parasites was determined at 72 h by the above-mentioned SYBR green I assay. The stage-specific parasite inhibition assay was performed in two independent experiments with technical triplicates each time.

#### 2.5.5. Asexual Parasite Growth Phenotype

To investigate whether treatment with the novkitasetaline could impair parasite growth during the intraerythrocytic development cycle (IDC), growth-related phenotypes were systematically determined, including growth curve, parasite multiplication rate, parasite egress, and invasion assays. Specifically, highly synchronized ring-stage parasites with ~6 h window were seeded at 0.1% parasitemia with 2% hematocrit in 24-well plate in the presence or absence of 1/4 × IC_50_, 1/2 × IC_50_, 1 × IC_50_, 2 × IC_50_, 4 × IC_50_ for 16 h at either early (0 ± 6 hpi) or late stage (24 ± 6 hpi). Novkitasetaline treatment was conducted one cycle prior to the phenotype assays. Following novkitasetaline treatment, cultures were washed twice with complete medium. Growth curves were plotted based on daily parasitemia assessed from Giemsa-stained thin smears. The multiplication rate of parasites was determined as the fold increase in parasitemia after each cycle. Three independent experiments with technical duplicates were performed for each experiment. Quantification of intraerythrocytic merozoites was conducted by microscopically counting 30 randomly selected schizont-infected erythrocytes.

For the egress-associated phenotype, the number of ruptured parasites was quantified following a standard protocol. Briefly, parasites were pre-treated with compounds for 24 h at the corresponding developmental stage prior to tight synchronization. Enriched schizonts by magnetic cell sorting were then co-incubated with red blood cells (RBCs) at 1% parasitemia and 2% hematocrit. After 12 h of incubation, the quantities of ring-stage trophozoites and schizonts were quantified by counting at least 5000 cells per experimental condition across randomly selected microscopic fields of Giemsa-stained thin blood smears. The number of ruptured schizonts and the average number of newly formed ring-stage parasites per ruptured schizont were calculated using the following formula: ruptured schizonts = [(parasitemia of schizont at 0 h) − (parasitemia of schizont at 12 h)]/(parasitemia of schizont at 0 h)

For the invasion-associated phenotype, the erythrocyte invasion assay was performed according to the previously reported protocol. Briefly, mature schizonts were purified by centrifugation on a 40/70% Percoll gradient, and enriched schizont-infected erythrocytes were counted by hemocytometer. 8 × 10^5^ schizonts were mixed with fresh erythrocytes at a 1:50 ratio in 200 μL complete medium. Mixtures were added into 96-well plates with triplicate wells for each treatment and incubated under standard conditions for an additional 20 h. The invasion rate was quantified by microscopic counting of newly invaded ring-stage parasites in at least 50 microscopic fields.

#### 2.5.6. Asexual Parasite Progression Assay

To analyze the arrested progression of parasites during the asexual stage, parasites were synchronized with two rounds of 5% sorbitol prior to assay. Synchronized ring-stage parasites were set up at 5% parasitemia, and the starting culture was divided into two aliquots. Half of the starting culture (ring stage parasites) was incubated with the compound at 2× IC_50_ in a 24-well plate to evaluate its effect on early-stage development. After 24 h, late-stage parasites progressed from the starting culture were similarly applied to evaluate the effect on late-stage development. After incubation, cultures were washed three times with complete medium to remove the compound, and the parasite progression was monitored during the following cycle. Thin blood smears were made at 4 h intervals, and microscopic analysis of growth arrest was performed by counting the number of newly invaded rings, as well as remaining trophozoites and schizonts in 100 parasites. The assay was performed in two independent experiments with technical duplicates each time.

## 3. Results

### 3.1. Activation of β-Carboline Alkaloid Biosynthesis in PRh3 by Ribosome Engineering

**Figure 1:**
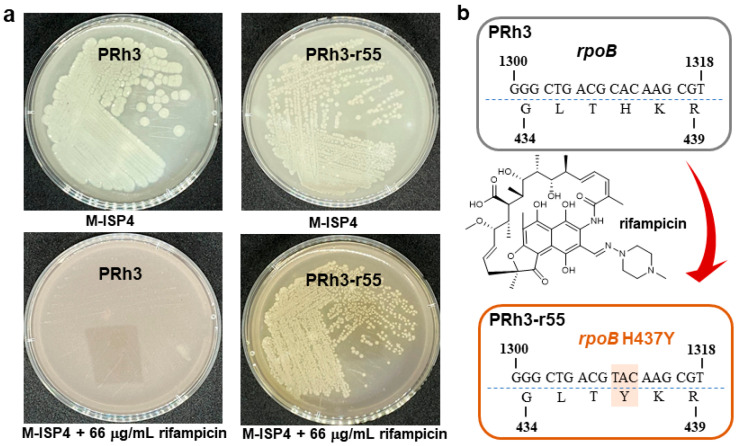
The comparison between PRh3 and mutant PRh3-r55. (**a**) The plate-cultured morphology of PRh3 and mutant PRh3-r55. (**b**) The mutant site confirmation of *rpoB* in PRh3-r55.

**Figure 2:**
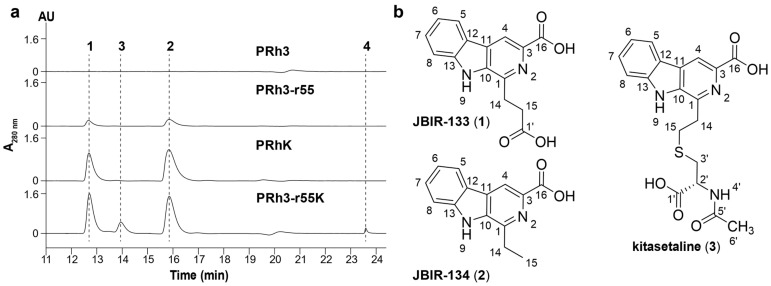
(**a**) HPLC analysis of PRh3 and its mutant strains. (**b**) Chemical structures of JBIR-133 (**1**), JBIR-134 (**2**), and kitasetaline (**3**).

DXWR, discovered in Dongxiang County, Jiangxi Province, China, is the northernmost-growing wild rice species in the world [14]. DXWR exhibits remarkable environmental adaptability and stress tolerance, which enables it to host a unique and rich collection of exploitable microbial strains [14,22,23]. PRh3 is a new *Streptomyces* strain isolated from DXWR, with promising potential for producing SMs (Figure 1a and Figure S1). Thus, subsequent fermentation and product analysis of PRh3 were conducted, but without any valuable SM signal being detected. This unexpected result was most likely due to the disparity between laboratory culture conditions and the natural habitat of PRh3, a distinct situation that frequently repressed BGCs expression in *Streptomyces* strains [5,6]. Thus, to address this challenge, we applied the simple and effective ribosome engineering to PRh3 by treating it with rifampicin at a concentration gradient. Hence, a series of rifampicin-resistant mutant strains was generated under corresponding rifampicin stress. Then, these mutant strains were further screened through strain fermentation and product analysis, which, in turn, selected the optimal mutant strain PRh3-r55 (Figure 1a). This mutant can produce two new products (**1** and **2**) compared with the WT control (Figure 2, line I–II). Meanwhile, genetic mutation site analysis of PRh3-r55 revealed it contained a typical H473Y mutation in the *rpoB*, thereby conferring tolerance to 66 µg/mL rifampicin (Figure 1b). This mutation is also frequently observed in other mutant *Streptomyces* strains generated via rifampicin-based ribosome engineering [9,10,11,12,13].

Through scale-up fermentation and product purification of PRh3-r55, **1** and **2** were identified as the β-carboline alkaloids JBIR-133 and JBIR-134, respectively (Figure 2 and Figures S6–S11 and Table 1) [16]. These results demonstrate that ribosome engineering successfully activated the biosynthetic potential of β-carboline alkaloids in PRh3, thereby providing the mutant strain PRh3-r55 for further development.

### 3.2. Heterologous Expression of ksl to Enhance β-Carboline Alkaloids Biosynthesis in PRh3-r55

**Figure 3:**
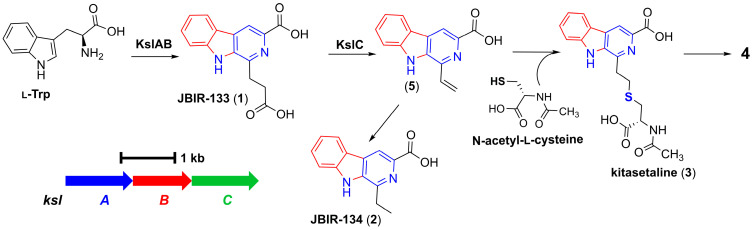
The biosynthetic mechanism of β-carboline alkaloids derived from *ksl*.

JBIR-133 and JBIR-134, previously isolated from *K. setae* NBRC 14216^T^, were biosynthesized by BGC *ksl* (Figure 3). However, we were unable to identify a BGC homologous to *ksl* in PRh3 due to the difficulties in purifying PRh3’s high-quality genomic DNA for complete genome sequencing. Meanwhile, considering the product consistency between PRh3-r55 and *K. setae* NBRC 14216^T^, we sought to express *ksl* in PRh3 and its mutant PRh3-r55. Thus, the intact *ksl* was cloned onto the vector containing gene expression elements SP44 + SR40 to construct the SP44 + SR40-*ksl* fusion, which could efficiently be expressed in *Streptomyces* strains [19]. Then, this BGC expression clone was introduced into PRh3 and PRh3-r55 to generate corresponding mutant strains PRh3K and PRh3-r55K, respectively.

Following comparison analysis revealed that the heterologous expression of *ksl* could effectively improve the β-carboline alkaloid biosynthesis and promoter PRh3K and PRh3-r55K to produce more JBIR-133 and JBIR-134 than PRh3-r55 (Figure 2, line III–IV). More importantly, with synergistic effects of ribosomal engineering-activated intracellular metabolism and *ksl* expression, PRh3-r55K not only acquired the highest yields of JBIR-133 and JBIR-134 but also simultaneously produced two new detected products **3** and **4** (Figure 2, line IV). ESI-HRMS analysis determined the molecular formula of **3** to be C_19_H_19_N_3_O_5_S (*m*/*z* 402.1114, [M + H]^+^) (Figure S12). Subsequent purification and NMR analysis confirmed **3** as kitasetaline (Figures S12–S14 and Table 1). Compound **4** was obtained in a considerably lower yield. ESI-HRMS analysis determined the molecular formula of **4** to be C_29_H_29_N_5_O_4_S (*m*/*z* 544.2007, [M + H]^+^) (Figure S15), which indicated that it also contains a sulfur atom, suggesting that **4** is likely a derivative of kitasetaline (Figures S12–S15 and Table 1). In contrast, the targeted comparative analysis did not detect any signals of kitasetaline and **4** in PRh3K and PRh3-r55 (Figure 2, line I–III). These results indicated that combining ribosome engineering with the heterologous expression of *ksl* is more effective than employing either method alone. This combinatorial strategy may optimize the intracellular metabolic environment of PRh3-r55K to facilitate the conversion of adequate intermediates to kitasetaline and product **4** (Figure 3).

### 3.3. Structure Elucidation of New β-Carboline Alkaloid Novkitasetaline

**Figure 4:**
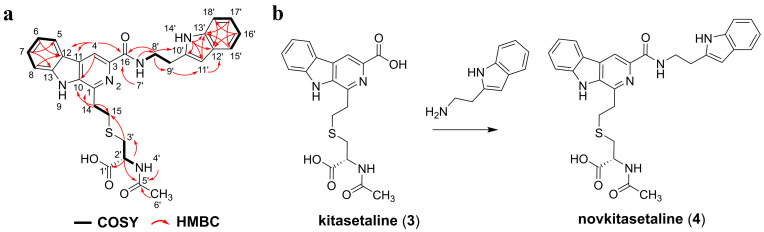
(**a**) Key COSY and HMBC correlations of novkitasetaline (**4**). (**b**) The modification of kitasetaline (**3**) to biosynthesize novkitasetaline (**4**).

The isolated purified product **4** was afforded as a pale yellow amorphous solid. Analysis of its ^1^H NMR indicated the existence of two methylene protons and five aromatic protons. The COSY correlations demonstrated spin systems: H-5 (δ 8.34)/H-6 (δ 7.28)/H-7 (δ 7.60–7.55)/H-8 (δ 7.68–7.62), indicating an ortho-disubstituted benzene ring skeleton; H-14a (δ 3.48–3.38)/H-14b (δ 3.48–3.38)/H-15 (δ 3.03); and H-3′a (δ 3.03)/H-3′b (δ 3.03)/H-2′ (δ 4.39)/4′-NH (δ 8.02). The HMBC spectrum of **4** showed the following long-range heteronuclear correlation signals: H-14a/C-10 (δ 136.04)/C-1 (δ 143.10), indicating that the C-14 (δ 34.52)–C-15 (δ 30.02) fragment is attached to C-1. The HMBC cross peaks from H-2′ to C-3′ (δ 33.54), C-1′ (δ 173.03), and C-5′ (δ 169.53), as well as from H-3′a, H-3′b to C-1′, and C-15, suggest the presence of a cysteine residue in **5**. Based on the key HMBC correlations from H-6′ (δ 1.87) to C-5′ and from H-2′ to C-5′, an N-acetyl group is located on the N-terminus of the cysteine residue [24]. This section was essentially consistent with the previously reported spectroscopic data of kitasetaline, which means the presence of a β-carboline-3-carboxylic acid moiety connected to N-acetylcysteine via a thioether linkage in **4** (Figure 4a and Figures S15–S20 and Table 1) [24].

Furthermore, two methylene resonances and five aromatic proton signals were also observed in the 1D spectrum of **4**. The COSY correlations revealed three partial structures: H-15′ (δ 7.68–7.62)/H-16′ (δ 6.99)/H-17′ (δ 7.08)/H-18′ (δ 7.35), along with the ^13^C NMR of H-11′ (δ 7.24) was observed as an isolated aromatic proton signal, suggesting the presence of a tryptophan residue. This is consistent with the correlations observed in the HMBC spectrum, such as 14′-NH (δ 10.92)/C-11′ (δ 123.17)/C-12′ (δ 127.75), H-11′/C-13′ (δ 136.80), H-15′/C-13′/C-17′ (δ 121.41), H-16′/C-12′/C-18′ (δ 111.87), H-17′/C-13′, H-18′/C-12′. The key HMBC correlations of H-9′ (δ 3.03)/C-11′, H-8′ (δ 3.66)/C-16 (δ 165.17)/C-10′ (δ 112.35), and 7′-NH (δ 8.73)/C-8′ (δ 40.04) confirmed the kitasetaline fragment is linked to a tryptamine through an amide bond (Figure 4 and Figures S15–S20 and Table 1). The optical rotation data of **4** was determined as [a]^25^_D_-8.6 (c 4.5, MeOH). This negative [a]_D_ value was also consistent with kitasetaline, which suggested that **4** had the absolute configuration of 2′*R* [24]. Taken together, **4** was identified as a novel β-carboline alkaloid and named as novkitasetaline (Figure 4 and Figures S15–S20, and Table 1). Obviously, novkitasetaline is a new derivative of kitasetaline (Figure 4b). The conversion of kitasetaline to novkitasetaline may be catalyzed by the amide synthetase to directly form an amide bond between the carboxyl group at C-3 of the pyridine ring in kitasetaline and a tryptamine (Figure 4b) [25]. This structural modification provides evidence that ribosome engineering remodeled the intracellular metabolic environment of the mutant PRh3-r55, endowing it with a broader repertoire of catalytic enzymes than that of the wild-type PRh3.

### 3.4. Novkitasetaline Exhibits In Vitro Antimalarial Activity Against Both Drug-Sensitive and Resistant Parasites

**Figure 5:**
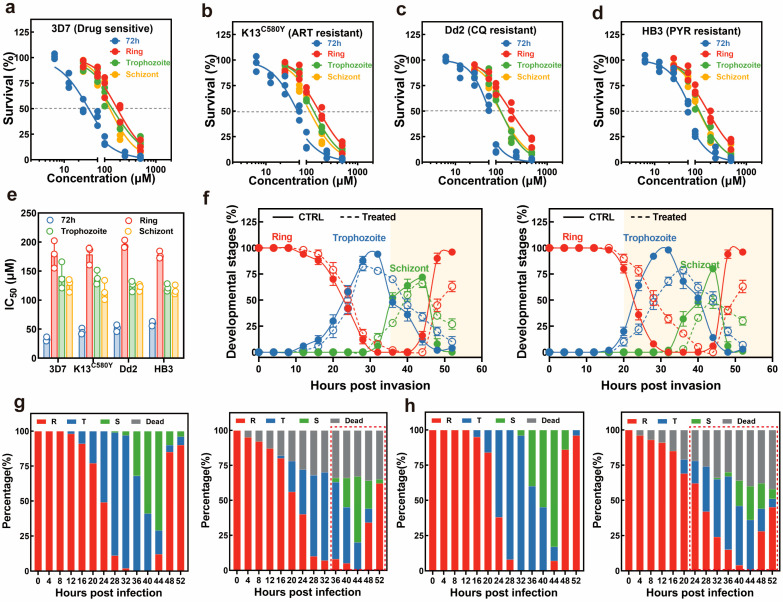
Effect of novkitasetaline on *P. falciparum* during intraerythrocytic development. Typical dose–response evaluations of novkitasetaline on asynchronous cultures of various *P. falciparum* strains during the asexual stage, including drug-sensitive 3D7 strain (**a**), artemisinin-resistant strain K13^C580Y^ (**b**), chloroquine-resistant Dd2 strain (**c**), and pyrimethamine-resistant HB3 strain (**d**). (**e**) IC_50_ values for different parasite strains subjected to either 72 h or 16 h stage-specific assays. (**f**) Comparison of the developmental progression of ring, trophozoite, and schizont stages throughout the IDC using the synchronized parasites pretreated with novkitasetaline at early/late stages. At each time point, the percentages of ring-stage (red), trophozoite-stage (blue), and schizont-stage (green) parasites are depicted. The solid line corresponds to the vehicle-treated group, and the dashed line corresponds to the novkitasetaline-treated group. The highlighted background corresponds to the differences in the parasite’s developmental stages after treatment. (**g**,**h**) Progression throughout the IDC growth cycle (encompassing the transition from ring to schizont stage) of novkitasetaline. The percentage of parasites (n = 100) exhibiting distinct morphological features after vehicle control treatment at the corresponding stages was assessed via microscopic examination (**g**,**h**, left panel). Similarly, the percentage of parasites (n = 100) displaying distinct morphological characteristics following novkitasetaline exposure at the corresponding stage was determined using microscopic examination. At each time point, parasites were morphologically classified into ring, trophozoite, schizont, or dead forms (**g**,**h**, right panel). The differences in the parasite’s developmental stages are circled with a red dashed line.

Previous research has verified that β-carboline alkaloids exhibited promising potential in antimalarial drug development [26,27,28]. Thus, we subsequently investigated the antimalarial activity of all isolated compounds. The in vitro antimalarial activity of compounds was evaluated against several *P. falciparum* strains with distinct drug sensitivity, including a wild-type (WT) drug-sensitive 3D7 strain, an artemisinin-resistant K13^C580Y^ strain, a chloroquine-resistant Dd2 strain, and a pyrimethamine-resistant HB3 strain. The inhibitory effect of novkitasetaline on asexual stage parasite proliferation was assessed using a 3-day growth inhibition assay. Representative growth inhibition curves are depicted in Figure 5, indicating that novkitasetaline potently suppressed the viability of drug-sensitive parasite strains with a 72 h IC_50_ of 32.65 ± 2.93 μM (Figure 5a,e and Table S4), while the JBIR-133, JBIR-134, and kitasetaline did not show obvious inhibition to the above parasite strains (IC_50_ > 200 mM) (Table S4). This result also confirmed that the modification of kitasetaline to biosynthesize novkitasetaline can significantly increase the compound’s antimalarial bioactivity. To explore the broader applicability of novkitasetaline against other *P. falciparum* strains, we subsequently determined its antimalarial activity against multiple drug-resistant strains (Figure 5b–d). The calculated IC_50_ values for each parasite strain ranged from 45.98 ± 4.17 to 59.67 ± 3.15 μM, with no statistically significant variation across strains (Figure 5b–e and Table S4). These findings have indicated that novkitasetaline was a new β-carboline alkaloid, can effectively inhibit *P. falciparum* growth regardless of the parasites’ drug resistance phenotypes.

### 3.5. Parasites Display Stage-Specific Susceptibility to Novkitasetaline

Owing to the unique developmental profile of *P. falciparum* during the intraerythrocytic life cycle, it has been validated that the parasite displays variations in sensitivity to common antimalarials [29]. Thus, such variability further represents a key potential mechanism driving the emergence of drug resistance in *Plasmodium*. Subsequently, to gain deeper insights into the stage-specific activity profile of novkitasetaline and thereby preliminarily delineate its mechanism of action, a stage-specific drug efficacy assay was conducted to identify the peak activity during the IDC. The growth inhibition profile of the parasite across different stages was illustrated in Figure 5b–d. At 72 h post-infection (hpi), all novkitasetaline-treated parasite populations exhibited a statistically significant reduction in survival rate relative to the untreated control. Notably, our results revealed that *Plasmodium* at the late trophozoite–schizont stages exhibited significantly greater sensitivity to novkitasetaline compared with parasites at the early ring stage (Figure 5e). This finding strongly suggests that novkitasetaline exerts its antimalarial activity primarily by targeting critical developmental processes of *P. falciparum* during the transition from trophozoite to schizont stages, aligning with the marked parasitemia reduction observed in these developmental phases.

### 3.6. Novkitasetaline Treatment Impairs Parasite Progression During IDC

To evaluate whether novkitasetaline pretreatment could alter parasite progression during the IDC, we monitored the temporal dynamics of distinct stages in synchronized parasite cultures at 4 h intervals (Figure 5f–h). Throughout the entire observation period, the growth profiles of parasites exposed to the novkitasetaline at either the early or late stage were altered, specifically regarding the transition rates from ring to trophozoite stages, as well as from trophozoite to schizont stages. Additionally, the duration of each developmental stage, including the trophozoite maturation (12–24 hpi) and schizont segmentation (24–48 hpi), was significantly prolonged, suggesting the novkitasetaline treatment could perturb the normal progression of the parasite.

### 3.7. Novkitasetaline Treatment Affects Parasite Proliferation

**Figure 6:**
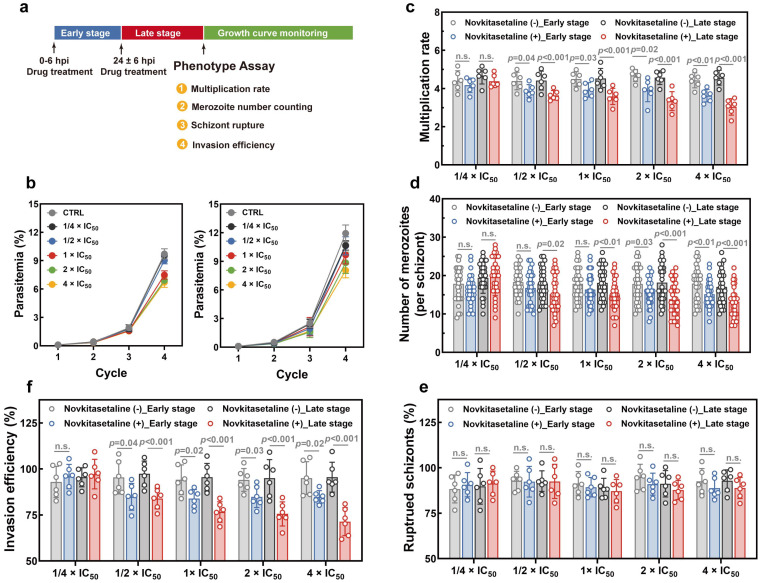
Effect of novkitasetaline on *P. falciparum* growth and proliferation. (**a**) Schematic illustration of the novkitasetaline pulse period and phenotype assays. (**b**) Asexual growth curve of 3D7 parasites cultured pretreated with novkitasetaline at early (left panel) and late stages (right panel) with different concentrations. (**c**) Parasite multiplication rate determination assay. The multiplication rate for parasites after novkitasetaline exposure was calculated as the fold increase in parasitemia per 48 h period. (**d**) Number of merozoites per schizont. The number of merozoites in each mature schizont was quantified by microscopically counting 30 randomly selected schizont-infected erythrocytes. (**e**) Comparison of the effects of novkitasetaline or vehicle treatment on parasite egress by counting the percentage of ruptured schizonts in the vehicle control group. (**f**) Comparison of the effects of novkitasetaline or vehicle treatment on the merozoite invasion. Data were presented as mean ± SEM from three independent experiments with technical duplicates. *p*-values were determined using Student’s *t*-test and n.s. corresponded to no statistical significance.

Then, phenotype assays have been performed to investigate whether the novkitasetaline treatment could affect parasite proliferation (Figure 6a). The parasite growth curve showed that exposure to novkitasetaline at the early stage led to a significant decrease in parasitemia at the 4th life cycle. In contrast, treatment at the late stage resulted in more pronounced growth inhibition. For late-stage exposure, parasitemia in the treated group significantly decreased starting from the third life cycle, and the inhibitory effect was dose dependent (Figure 6b). These results preliminarily indicate that novkitasetaline treatment can affect parasite growth and proliferation during the IDC.

In order to detailly characterize the effect of novkitasetaline on parasite development, the multiplication rate was further quantified based on the parasitemia at subsequent cycles. As shown in Figure 6c, the vehicle control group exhibited a parasite multiplication rate of ~4.5. In contrast, parasites exposed to novkitasetaline (either at the early or late stage) showed significantly reduced multiplication rates at concentrations above 1/2 × IC_50_. Consistent with the growth curve data, novkitasetaline exerted a more pronounced effect on late-stage parasites, with a clear dose-dependent reduction in multiplication rate observed. While early-stage exposure also impaired parasite multiplication, this inhibitory effect was less prominent and lacked a consistent dose–response relationship compared to late-stage treatment. Furthermore, pretreatment with the novkitasetaline at the late stage substantially impaired parasite schizogony based on a decrease in the number of merozoites per schizont (Figure 6d). To determine whether the impaired parasite proliferation stemmed from defects in egress or invasion, a comprehensive phenotypic analysis was performed. Results from the schizont egress assay indicated that parasite egress was minimally affected by novkitasetaline exposure (either at the early or late stage), as reflected by the percentage of ruptured schizonts per cycle (Figure 6e). This finding further confirms that the reduced parasitemia was not attributed to impaired merozoite release. Additionally, a significant, dose-dependent reduction in invasion efficiency was observed following novkitasetaline treatment at the late stage. This suggests that the antimalarial effect of novkitasetaline arises from both impaired parasite growth and reduced merozoite invasion (Figure 6f).

In summary, these results indicate that novkitasetaline treatment at the late stage can lead to impaired IDC development in parasites, which predominantly impacts schizogony and the invasion process rather than egress.

## 4. Discussion

The deep exploration of the rich bioactive SMs from *Streptomyces* is a major objective in current drug development [1,2,3,4]. However, this exploration is always hindered due to the severe disparity between laboratory culture conditions and the natural habitats of *Streptomyces* strains, which in turn can suppress the expression of BGCs within strains and halt the production of SMs [5]. Directly altering culture conditions to bridge the gap of strain growth involves manipulating numerous critical physical and chemical parameters, which is difficult to achieve in standard cultivation protocols. To circumvent these limitations and facilitate the SMs discovery, ribosome engineering was developed and applied in *Streptomyces* research [9,10,11,12,13]. This strategy is first performed by only selecting common laboratory antibiotics that target either the RNAP or the ribosome itself [12]. Antibiotics such as rifampicin can be seamlessly integrated into conventional strain cultivation methods, without significantly altering medium composition or adversely affecting the cultivation operation [12,13]. Furthermore, all suitable antibiotics can be used quantitatively via concentration gradients in the medium to treat the target strains, thereby generating resistant mutants [9,10,11,12]. Thus, this overall procedure of ribosome engineering is relatively simple and convenient.

On the other hand, compared to traditional mutagenesis strategies, ribosome engineering has more defined targets: RNAP and ribosome, which are central to gene expression [9,10,11,12,13]. The resulting antibiotic-resistant mutant strains of ribosome engineering always acquire mutations in RNAP or ribosomal proteins, leading to global adjustments in intracellular gene expression and activating the expression of silent BGCs [6,9,10,11,12,13]. Finally, the optimized BGCs expression could initiate and enhance the production of SMs [6,9,10,11,12,13]. Given these superior traits of ribosome engineering, it has been extensively employed in the research and exploitation of *Streptomyces*, facilitating the discovery of diverse notable bioactive lead compounds [6,9,10,11,12,13]. In this research, to efficiently elicit the production of SMs in PRh3 under laboratory conditions, we treated it only with rifampicin, a common antibiotic used in ribosome engineering. These processes led to the screening of the optimal mutant strain PRh3-r55, which effectively produced JBIR-133 and JBIR-134. This activation of β-carboline alkaloids biosynthesis in DXWR-derived PRh3 also further validated that ribosome engineering was a simple but effective strategy in *Streptomyces* SM development. Crucially, ribosome engineering can significantly enhance the efficiency and scope of both research and utilization of *Streptomyces* strains, thereby enabling the exploitation of numerous *Streptomyces* strains isolated from natural habitats [12,13,14,22,23]. Moreover, the diverse mutants generated by ribosome engineering, with upgraded intracellular BGC expression and improved SM profiles, may also provide new foundations to drive the continuous investigation.

Meanwhile, heterologous expression serves as another valuable strategy in *Streptomyces* SM development [30,31]. This strategy capitalizes on the continual progress in efficiently refactoring BGCs into amenable hosts, ultimately leading to successful BGC expression for biosynthesizing various SMs [30,31]. In the present research, unexpected difficulties in genome sequencing prevented the identification of β-carboline alkaloid BGC in PRh3. Consequently, we employed an alternative strategy by cloning the *ksl*, responsible for JBIR-133 and JBIR-134 biosynthesis, and introducing it into PRh3-r55 for heterologous expression. The efficient expression of *ksl*, coupled with ribosome engineering-improved intracellular environment in PRh3-r55, created a synergistic and iterative effect. This effect not only significantly enhanced the yield of JBIR-133 and JBIR-134, but also enabled the production of two sulfur-containing β-carboline alkaloids, including the new molecule novkitasetaline. These combinatorial practices underline that utilizing metabolically engineered hosts in conjunction with well-characterized BGCs can effectively facilitate the discovery of new NPs. Recently, a tremendous diversity of BGCs has been continuously identified from *Streptomyces* [1,2,3,4,5,6]. In parallel, ribosome engineering can steadily generate various *Streptomyces* hosts [9,10,11,12]. Therefore, integrating these two strategies can expand the scope for BGCs expression, enabling the deep mining of natural products and ensuring the full utilization of genetic resources.

β-carboline alkaloids, featuring a planar tricyclic pyridoindole ring system, constitute an extraordinary family of N-heterocyclic compounds widely distributed in plants and microorganisms [26,27]. Natural β-carboline alkaloids have exhibited potent antimalarial effects against diverse *Plasmodium* strains [26,27,28]. Thus, isolation and identification of new β-carboline alkaloids not only directly provide candidate antimalarial lead compounds, but also offer unique structure features to guide subsequent rational drug design and modification. In the present study, we obtained the new sulfur-containing antimalarial novkitasetaline, which exhibited a relatively broad antimalarial activity against both drug-sensitive and drug-resistant *P. falciparum* strains. Moreover, differences in drug sensitivity among developmental stages of *P. falciparum* have been reported as one of the potential mechanisms for the emergence of antimalarial drug resistance [29]. Thus, additional quantitative assessments of stage-specific drug sensitivity have been performed to investigate the mode of action (MOA) of novkitasetaline. Notably, in contrast to the 72 h growth inhibition assay, a similar growth profile as well as IC_50_ values have been obtained regardless of the *P. falciparum* strains. Late-stage parasites exhibit significantly higher sensitivity than early-stage parasites. Given that late-stage parasite proliferation is associated with the progression of the parasite’s IDC, this finding strongly indicates that exposing late-stage parasites to novkitasetaline can markedly impair parasite normal developmental processes. Detailed growth phenotype assays have confirmed that exposure to novkitasetaline at the late developmental stage significantly halts the parasite’s progression, in which both a notable reduction in the number of newly invaded ring-stage parasites and an increase in the accumulation of arrested schizont-stage parasites have been observed. Meanwhile, the reduced number of merozoites per schizont further indicated that exposure at the late stage can remarkably disrupt parasite development. Furthermore, invasion/egress assays provided additional support for this conclusion, confirming that novkitasetaline exposure impairs parasite development rather than interfering with schizont rupture. Collectively, our results demonstrated that novkitasetaline exerts its antimalarial activity by targeting *Plasmodium* growth processes.

Traditional antimalarials are classified as fast-acting drugs that can eliminate the vast majority of parasites within a short period. For example, artemisinin and its derivatives generate reactive oxygen species upon activation, which exerts a lethal effect on parasites [32]. Additionally, quinoline antimalarials act by inhibiting hemoglobin-degrading enzymes, thereby leading to the accumulation of toxic heme that ultimately kills the parasite [33]. However, due to the relatively high selection pressure imposed by their mode of action, these agents are prone to developing drug resistance [34]. It has been reported that targeting key transcriptional factors or proteins associated with parasite progression represents a promising strategy for developing novel antimalarials with reduced propensity for resistance evolution [35]. Among potential targets, tubulins, a core component of microtubules, have emerged as possible candidates for inhibiting parasite growth [36,37]. Disruption of tubulin function, whether via destabilizing or stabilizing compounds, triggers parasite growth arrest or developmental impairment. Notably, several alkaloid compounds (e.g., colchicine alkaloids and vinca alkaloids) have been identified as tubulin destabilizers [38]. Given the structural similarity between novkitasetaline and these alkaloids, we hypothesize that novkitasetaline exerts its antimalarial effect by disrupting tubulin function. Our results further support this hypothesis; for instance, parasite growth was more significantly impaired when exposed to novkitasetaline at the late stage. This is consistent with the fact that late-stage parasites undergo extensive morphological changes, during which microtubules play a critical role in forming and maintaining the mitotic spindle, an essential structure for chromosome segregation during cell division [39]. Additionally, unlike compounds that interfere with parasite egress (e.g., ML10, E64), novkitasetaline only reduced the parasite multiplication rate and inhibited schizogony rather than blocking egress [40,41]. Notably, direct evidence linking novkitasetaline to tubulin targeting is currently lacking. This is partly due to its moderate antimalarial potency (weaker than traditional antimalarials) and the absence of commercially available *Plasmodium* tubulin-specific antibodies.

While novkitasetaline exhibits relatively weaker antimalarial activity compared to commercially available antimalarials, its notable efficacy against drug-resistant parasite strains highlights its potential as a candidate partner drug in combating malaria drug resistance. This property positions it as a valuable starting point for addressing the critical challenge of resistance in current antimalarial therapies. To advance its potential, future research could focus on two key directions based on its structural scaffold, including the structural optimization and in vivo antimalarial evaluation. Guided by structure–activity relationship analyses, targeted modifications could be further implemented to improve its antimalarial potency. Based on these efforts, in vivo studies including assessments of pharmacokinetic profiles, efficacy in different animal models of malaria could be conducted to validate their translational potential. Furthermore, the novkitasetaline is a modified natural derivative of kitasetaline. The distinct structural features and bioactivity between these two compounds definitively validate that aromatic modification at the C-1 position of the β-carboline pyridine ring is critical for antimalarial efficacy. This structure-activity relationship aligns with earlier findings on marinacarbolines [25,28], offering valuable insights and reference for future antimalarial drug design and development.

## 5. Conclusions

In summary, the dormant β-carboline alkaloid biosynthesis pathway in DXWR-derived PRh3 was first activated via ribosome engineering to generate the optimal mutant strain PRh3-r55, which can steadily produce JBIR-133 and JBIR-134. Then, the intact BGC *ksl* was introduced into PRh3-r55 to generate the subsequent mutant strain PRh3-r55k for effective heterologous expression. These sequential strategies synergistically enhanced the β-carboline alkaloid biosynthesis of PRh3-r55k by producing the three theoretical products (JBIR-133, JBIR-134, and kitasetaline) as well as the novel compound, novkitasetaline. Novkitasetaline is a derivative of kitasetaline with a tryptamine moiety attached at the C-3 position of its pyridine ring. This structural modification confers antimalarial activity of novkitasetaline against both drug-sensitive and drug-resistant *P. falciparum* strains by targeting their growth processes. More importantly, novkitasetaline can significantly disrupt the normal developmental processes of IDC, a mechanism that confers significant potential for its application as a partner drug in combination therapeutic regimens. Therefore, this work confirmed that ribosome engineering coupled with heterologous expression can be effectively applied in the mining of *Streptomyces* strains SMs. The identified bioactive novkitasetaline can also serve as a promising lead compound for guiding antimalarial drug research and development.

## Figures and Tables

**Table 1:** NMR data of compounds.

Position	JBIR-133 (1)		JBIR-134 (2)		Kitasetaline (3)		Novkitasetaline (4)	
	δ_C_, Type	δ_H_, Multi (J/Hz)	δ_C_, Type	δ_H_, Multi (J/Hz)	δ_C_, Type	δ_H_, Multi (J/Hz)	δ_C_, Type	δ_H_, Multi (J/Hz)
1	144.16, C		146.98, C		144.08, C		143.10, C	
2								
3					136.34, C			
4	116.38, CH	8.85, s	116.00, CH	8.77, s	115.77, CH	8.73, s	112.61, CH	8.69, s
5	122.80, CH	8.40, d (7.9)	122.53, CH	8.36, d (7.9)	122.34, CH	8.32, d (7.9)	122.48, CH	8.34, d (7.9)
6	120.90, CH	7.34, t (7.4)	120.55, CH	7.30, td (7.4, 1.1)	120.05, CH	7.27, m	120.24, CH	7.28, t (7.5)
7	128.33, CH	7.63, t (7.6)	127.84, CH	7.59, ddd (8.2, 6.9, 1.2)	128.10, CH	7.57, ddd (8.2, 7.0, 1.2)	128.72, CH	7.60–7.55, m
8	112.91, CH	7.69, d (8.2)	112.75, CH	7.66, d (8.2)	113.05, CH	7.67, t (7.2)	112.79, CH	7.68–7.62, m
9								
10	136.18, C		135.87, C				136.04, C	
11	129.35, C		128.84, C		128.54, C		128.39, C	
12	121.67, C		121.83, C		121.71, C		121.83, C	
13	141.67, C		141.28, C		141.96, C		141.55, C	
14a	28.12, CH_2_		27.11, CH_2_	3.19, q (7.5)	35.90, CH_2_	3.57, td (12.4, 5.4)	34.52, CH_2_	3.48–3.38, m ^a^
14b						3.37, td (12.1, 5.0)		3.48–3.38, m ^a^
15	31.91, CH_2_	2.94, t (7.5)	13.12, CH_3_	1.40, t (7.5)	29.75, CH_2_	3.11, m	30.02, CH_2_	3.03, tq (12.6, 7.0)
16	166.62, C		167.31, C		167.92, C		165.17, C	
1′	174.52, C				173.56, C		173.03, C	
2′					54.11, CH	4.22, dt (7.0, 4.8)	53.30, CH ^b^	4.39, q (6.4)
3′a					33.22, CH_2_	2.78, ddd (13.3, 11.3, 5.1)	33.54, CH_2_	3.03, tq (12.6, 7.0)
3′b						3.11, m		3.03, tq (12.6, 7.0)
4′						7.67, t (7.2)		8.02, d (7.4)
5′					168.85, C		169.53, C	
6′					23.37, CH_3_	1.88, s	23.07, CH_3_	1.87, s
7′								8.73, t (6.0)
8′							40.04, CH_2_	3.66, q (7.0)
9′							25.90, CH_2_	3.03, tq (12.6, 7.0)
10′							112.35, C	
11′							123.17, CH	7.24, d (2.3)
12′							127.75, C	
13′							136.80, C	
14′								10.92, s
15′							118.92, CH	7.68–7.62, m
16′							118.69, CH	6.99, t (7.4)
17′							121.41, CH	7.08, t (7.5)
18′							111.87, CH	7.35, d (8.1)
16-OH				12.02, s				

^a^ Overlapped by water peaks. ^b^ Indicated in HSQC.

## Data Availability

The original contributions presented in this study are included in the article/Supplementary Materials. Further inquiries can be directed to the corresponding authors.

## Supplementary Materials

The following supporting information can be downloaded at: https://www.mdpi.com/article/10.3390/microorganisms13122871/s1. Figure S1: Phylogenetic analysis of PRh3; Figures S2–S5: Structure of JBIR-133, JBIR-134, kitasetaline and novkitasetaline; Figures S6–S8: The ESI-HRMS and NMR of JBIR-133; Figures S9–S11: The ESI-HRMS and NMR of JBIR-134; Figures S12–S14: The ESI-HRMS and NMR of kitasetaline; Figures S15–S20: The ESI-HRMS and NMR of novkitasetaline; Table S1: The primers used in identification of the mutation in *rpoB* from PRh3-r55; Table S2: The oligonucleotides used in construction of pJN44; Table S3: The primers used in amplification of *ksl*; Table S4: Antimalarial activities of compounds.
