# Supplementary material for: Mining of Novkitasetaline, a New Sulfur-Containing Antimalarial β-Carboline Alkaloid, from Streptomyces sp. PRh3 by Functional Ribosome Engineering Directed Heterologous Expression
Source: Microorganisms. 2025 Dec 18;13(12):2871. doi: 10.3390/microorganisms13122871 (PMC12735770; doi:10.3390/microorganisms13122871)

microorganisms

Article

Mining of Novkitasetaline, a New Sulfur-Containing Anti-malarial β-Carboline Alkaloid, from *Streptomyces* sp. PRh3 by Ribosome Engineering Directed Heterologous Expression

Xingyu Chen [1,†], Xiaohui He [2,†], Yanmin Wang [1], Yangping Feng [1], Zihan Wang [1], Chunhui Song [1] Xinyu Yu [2,3]* and Yunchang Xie [1,*]

1 Key Laboratory of Biodiversity Conservation and Bioresource Utilization of Jiangxi Province, College of Life Sciences, Jiangxi Normal University, Nanchang 330022, China

2 National Health Commission Key Laboratory of Parasitic Disease Control and Prevention, Jiangsu Provincial Key Laboratory on Parasite and Vector Control Technology, Jiangsu Institute of Parasitic Diseases, Wuxi, China

3 Center for Global Health, School of Public Health, Nanjing Medical University, Nanjing, China

* Correspondence: xieyunchang@jxnu.edu.cn; xinyu_yu@jipd.com.

† These authors contributed equally to this work.

Contents

Table S1	The primers used in identification of the mutation in *rpoB* from PRh3-r55	3
Table S2	The oligonucleotides used in construction of pJN44	3
Table S3	The primers used in amplification of *ksl*	3
Table S4	Anti-malarial activities of compounds	3
Figure S1	Phylogenetic analysis of PRh3	4
Figure S2	Structure of JBIR-133	5
Figure S3	Structure of JBIR-134	5
Figure S4	Structure of kitasetaline	5
Figure S5	Structure of novkitasetaline	6
Figure S6	ESI-HRMS spectrum of JBIR-133	6
Figure S7	^{1}H NMR (700 MHz) spectrum of JBIR-133 in DMSO	7
Figure S8	^{13}C NMR (175 MHz) spectrum of JBIR-133 in DMSO	7
Figure S9	ESI-HRMS spectrum of JBIR-134	8
Figure S10	^{1}H NMR (400 MHz) spectrum of JBIR-134 in DMSO	8
Figure S11	^{13}C NMR (100 MHz) spectrum of JBIR-134 in DMSO	9
Figure S12	ESI-HRMS spectrum of kitasetaline	9
Figure S13	^{1}H NMR (400 MHz) spectrum of kitasetaline in DMSO	10
Figure S14	^{13}C NMR (125 MHz) spectrum of kitasetaline in DMSO	10
Figure S15	ESI-HRMS spectrum of novkitasetaline	11
Figure S16	^{1}H NMR (600 MHz) spectrum of novkitasetaline in DMSO	11
Figure S17	^{13}C NMR (150 MHz) spectrum of novkitasetaline in DMSO	12
Figure S18	^{1}H-^{1}H COSY NMR spectrum of novkitasetaline in DMSO	12
Figure S19	^{1}H-^{13}C HMBC NMR spectrum of novkitasetaline in DMSO	13
Figure S20	^{1}H-^{13}C HSQC NMR spectrum of novkitasetaline in DMSO	13

Table S1. The primers used in identification of the mutation in *rpoB* from PRh3-r55

Sequence (5'-3')	Amplified products
**rpoB1-Fr**: TTGGCCGCCTCGCGCAACGC<br>**rpoB1-Re**: CCGCCGAGCTTCTTGTTCAC	928 bp
**rpoB2-Fr**: CGACCTCGCCAAGGTCGGCC<br>**rpoB2-Re**: CGGTGCCGACGAGCGGGGCC	962 bp
**rpoB3-Fr**: GGCCGTGCCGCTGATTAAGT<br>**rpoB3-Re**: GGATGATGTCGACCGGGGTC	908 bp
**rpoB4-Fr**: CGAGGACATGCCGTTCCTCG<br>**rpoB4-Re**: TCAGACCTCTTCGACGCTGC	826 bp

Table S2. The (two sequence complementary) oligonucleotides used in construction of pJN44.

Sequence (5'-3')
aattcTGTTCACATTCGAACCGTCTCTGCTTTGACAACATGCTGTGCGGTGTTGTAAAGTCTGGTGTAGGA<br>GAATACGACAGCTTGTCAAAGGAGTGTC**CATATGGGATCC**t
ctaga**GGATCCCATATG**GACACTCCTTTGACAAGCTGTCGTATTCTCCTACACCAGACTTTACAACACCG<br>CACAGCATGTTGTCAAAGCAGAGACGGTTCGAATGTGAACAg

The lowercases represent the cohesive ends; The bold capitalized letters represent the NdeI/BamHI sites

Table S3. The primers used in amplification of *ksl*

Sequence (5'-3')
**Ksl-Fr**: cttgtcaaaggagtgtccatATGGAGGGGACAGTGAAAACCGTAG
**Ksl-Re**: ctgcaggtcgactctagaggatccTCAGGAGACGGCGGTCTTCGACGGCG

Table S4. Anti-malarial activities of compounds

Compound	IC_{50}(μM)			
	3D7[a]	K13[C580Y,b]	Dd2[c]	HB3[d]
JBIR-133	>200	>200	>200	>200
JBIR-134	>200	>200	>200	>200
kitasetaline	>200	>200	>200	>200
novkitasetaline	32.65 ± 2.93	45.98 ± 4.17	51.88 ± 4.76	59.67 ± 3.15
chloroquine[e]	0.0115 ± 0.0021	0.0163 ± 0.0019	0.2988 ± 0.0361	0.1133 ± 0.0111

[a]Drug-sensitive line.[b]Artemisinin-resistant line.[c]Chloroquine-resistant line.

[d]Pyrimethamine-resistant line.[e]Positive control.

Figure S1. Phylogenetic analysis of PRh3.

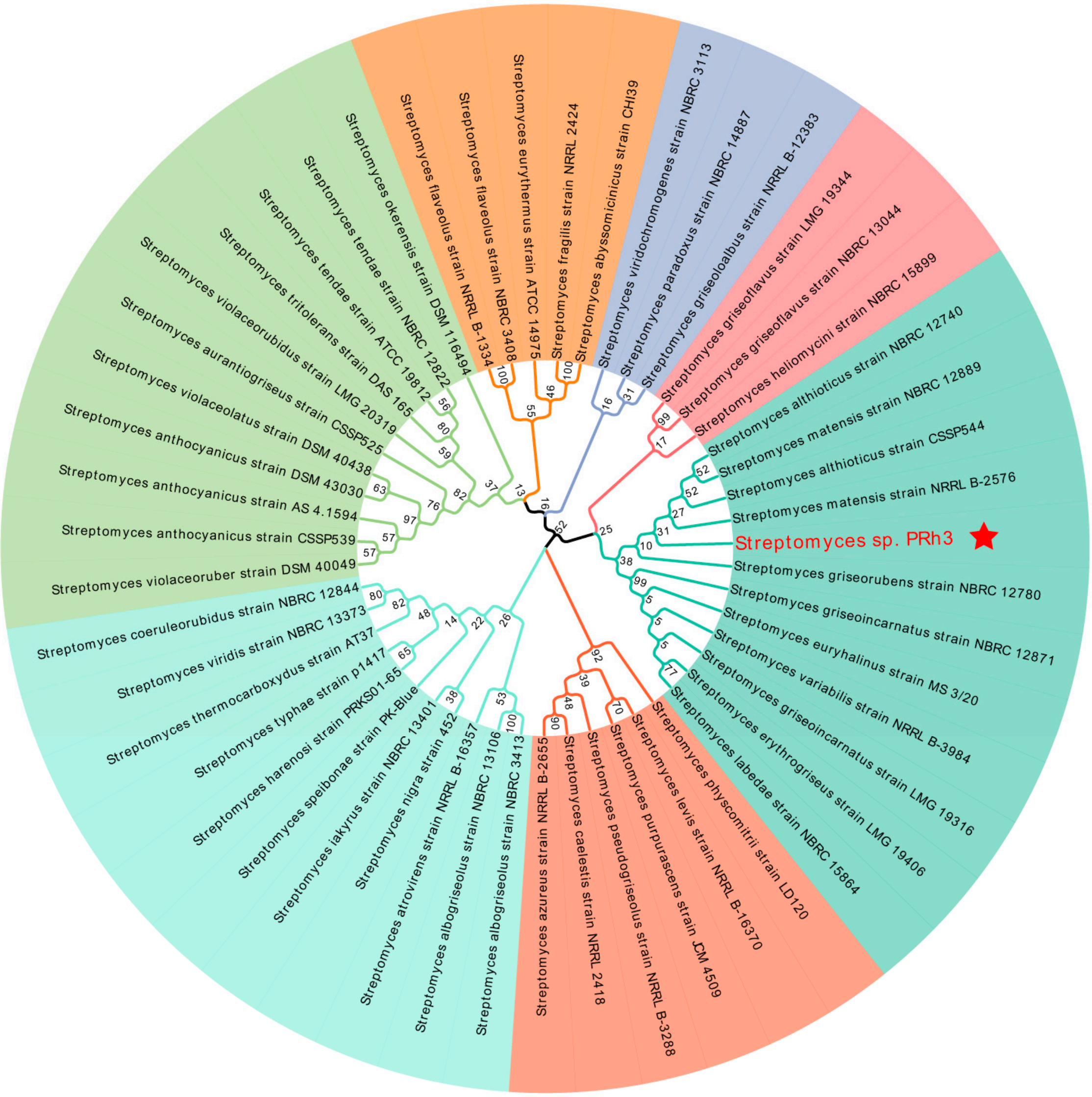

Figure S2. Structure of JBIR-133.

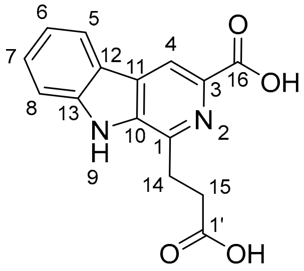

JBIR-133

JBIR-133; Pale yellow amorphous solid; ESI-HRMS *m/z* 285.0871 $[M+H]^+$ (calculated for $C_{15}H_{13}N_2O_4^+$, 285.0870); ^{13}C NMR(150 MHz, DMSO-d_6): δ 174.52, 166.62, 144.17, 141.67, 136.18, 129.35, 128.33, 122.80, 121.67, 120.90, 116.38, 112.91, 31.91, 28.12; ^1H NMR(700 MHz, DMSO-d_6): δ 8.85 (s, 1H), 8.40 (d, J = 7.9 Hz, 1H), 7.69 (d, J = 8.2 Hz, 1H), 7.63 (t, J = 7.6 Hz, 1H), 7.34 (t, J = 7.4 Hz, 1H), 2.94 (t, J = 7.5 Hz, 2H).

Figure S3. Structure of JBIR-134.

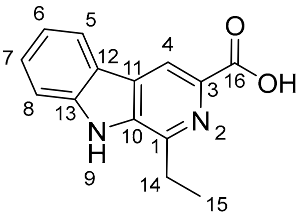

JBIR-134

JBIR-134; Pale yellow amorphous solid; ESI-HRMS *m/z* 241.0970 $[M+H]^+$ (calculated for $C_{14}H_{13}N_2O_2^+$, 241.0972). ^{13}C NMR(100 MHz, DMSO-d_6): δ 167.31, 146.98, 141.28, 137.02, 135.87, 128.84, 127.84, 122.53, 121.83, 120.55, 116.00, 112.75, 27.11, 13.12; ^1H NMR(400 MHz, DMSO-d_6): δ 12.02 (s, 1H), 8.77 (s, 1H), 8.36 (d, J = 7.9 Hz, 1H), 7.66 (d, J = 8.2 Hz, 1H), 7.59 (ddd, J = 8.2, 6.9, 1.2 Hz, 1H), 7.30 (td, J = 7.4, 1.1 Hz, 1H), 3.19 (q, J = 7.5 Hz, 2H), 1.40 (t, J = 7.5 Hz, 3H).

Figure S4. Structure of kitasetaline.

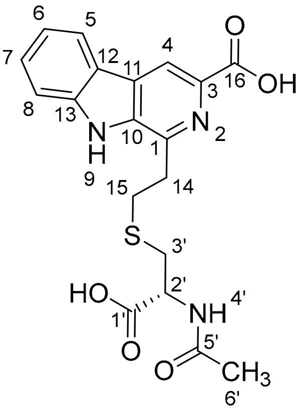

kitasetaline

Kitasetaline; Pale yellow amorphous solid; ESI-HRMS *m/z* 402.1114 $[M+H]^+$ (calculated for $C_{19}H_{20}N_3O_5S^+$, 402.1118). ^{13}C NMR(125 MHz, DMSO-d_6): δ 173.56, 168.85, 167.92, 144.08, 141.96, 136.34, 128.54, 128.10, 122.34, 121.71, 120.05, 115.77, 113.05, 54.11, 35.90, 33.22, 29.75, 23.37; ^1H NMR(400 MHz, DMSO-d_6): δ 8.73 (s, 1H), 8.32 (d, J = 7.9 Hz, 1H), 7.67 (t, J = 7.2 Hz, 2H), 7.57 (ddd, J = 8.2, 7.0, 1.2 Hz, 1H), 7.33–7.22 (m, 1H), 4.22 (dt, J = 7.0, 4.8 Hz, 1H), 3.57 (td, J = 12.4, 5.4 Hz, 1H), 3.37 (td, J = 12.1, 5.0 Hz, 1H), 3.23–2.99 (m, 3H), 2.78 (ddd, J = 13.3, 11.3, 5.1 Hz, 1H), 1.88 (s, 3H).

Figure S5. Structure of novkitasetaline.

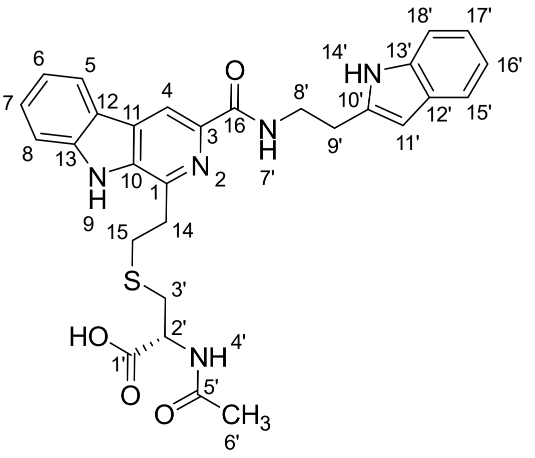

novkitasetaline

Novkitasetaline; Pale yellow amorphous solid; ESI-HRMS *m/z* 544.2007 $[M+H]^+$ (calculated for $C_{29}H_{30}N_5O_4S^+$, 544.2013). ^{13}C NMR(150 MHz, DMSO-d_6): δ 169.53, 165.17, 143.10, 141.55, 136.80, 136.04, 128.39, 127.75, 123.17, 122.48, 121.83, 121.41, 120.24, 118.92, 118.69, 112.79, 112.61, 112.35, 111.87, 33.54, 30.02, 25.90, 23.07; ^1H NMR(600 MHz, DMSO-d_6): δ 10.92 (s, 1H), 8.73 (t, J = 6.0 Hz, 1H), 8.69 (s, 1H), 8.34 (d, J = 7.9 Hz, 1H), 8.02 (d, J = 7.4 Hz, 1H), 7.68–7.62 (m, 2H), 7.60–7.55 (m, 1H), 7.35 (d, J = 8.1 Hz, 1H), 7.28 (t, J = 7.5 Hz, 1H), 7.24 (d, J = 2.3 Hz, 1H), 7.08 (t, J = 7.5 Hz, 1H), 6.99 (t, J = 7.4 Hz, 1H), 4.39 (q, J = 6.4 Hz, 1H), 3.66 (q, J = 7.0 Hz, 2H), 3.03 (tq, J = 12.6, 7.0 Hz, 6H), 1.87 (s, 3H).

Figure S6. ESI-HRMS spectrum of JBIR-133.

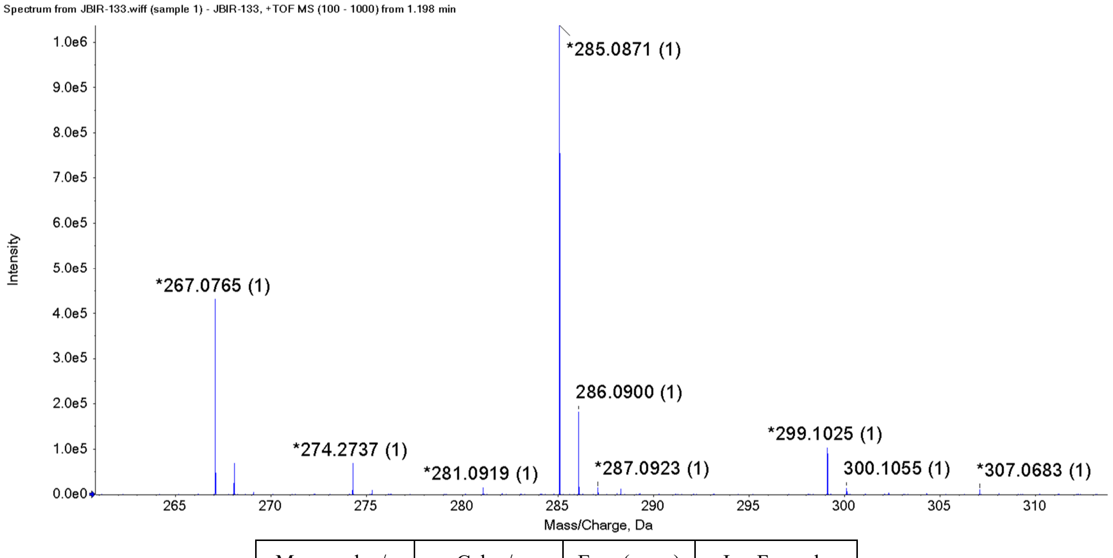

Measured *m/z*	Cal *m/z*	Error(mmu)	Ion Formula
285.0871	285.0870	0.1	$C_{15}H_{13}N_2O_4^+$

Figure S7. ^{1}H NMR (700 MHz) spectrum of JBIR-133 in DMSO.

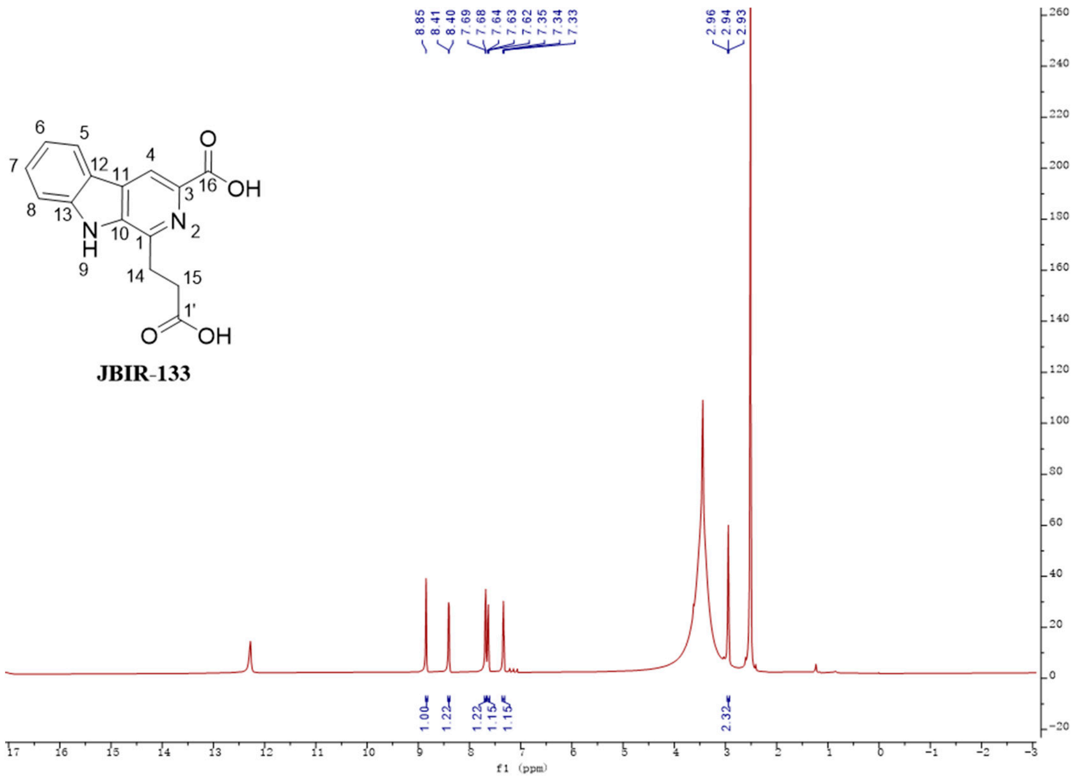

Figure S8. ^{13}C NMR (175 MHz) spectrum of JBIR-133 in DMSO.

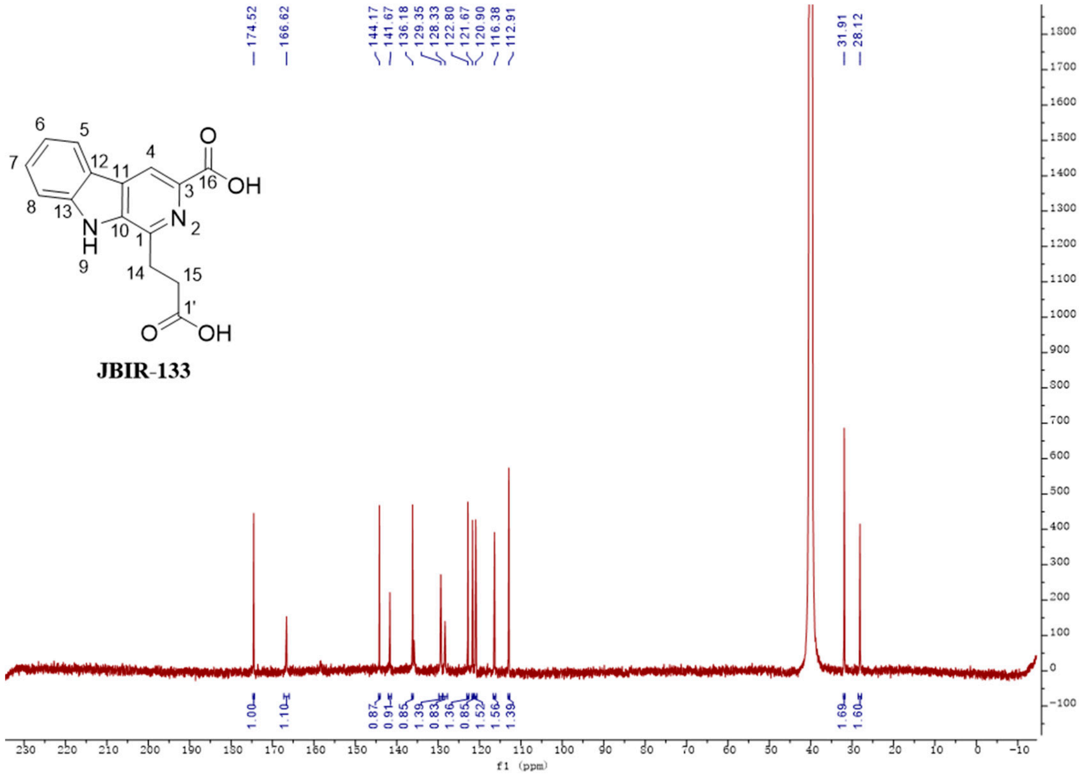

Figure S9. ESI-HRMS spectrum of JBIR-134.

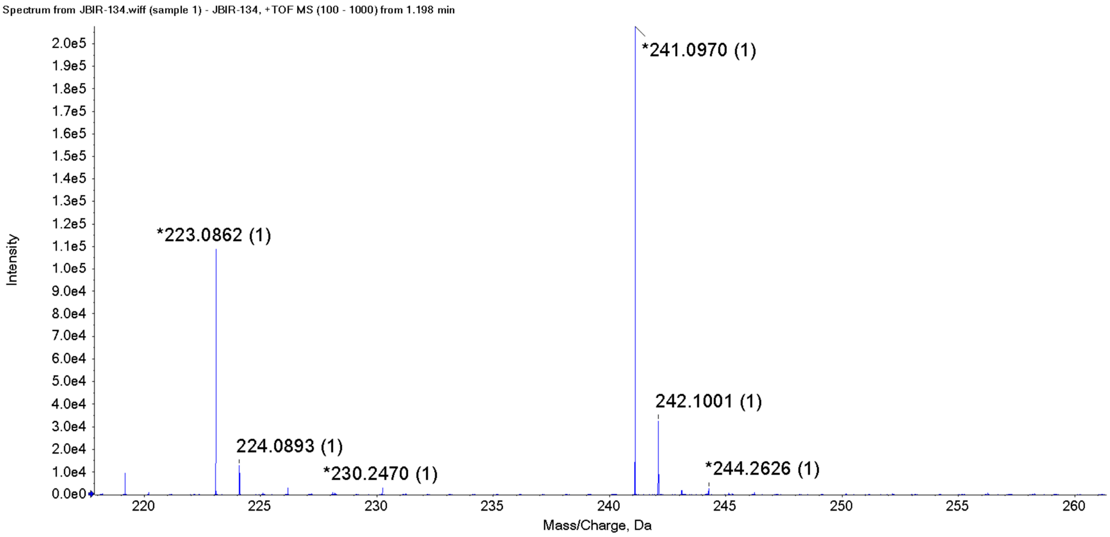

Measured *m*/*z*	Cal *m*/*z*	Error(mmu)	Ion Formula
241.0970	241.0972	-0.2	$C_{14}H_{13}N_2O_2^+$

Figure S10. ^{1}H NMR (400 MHz) spectrum of JBIR-134 in DMSO.

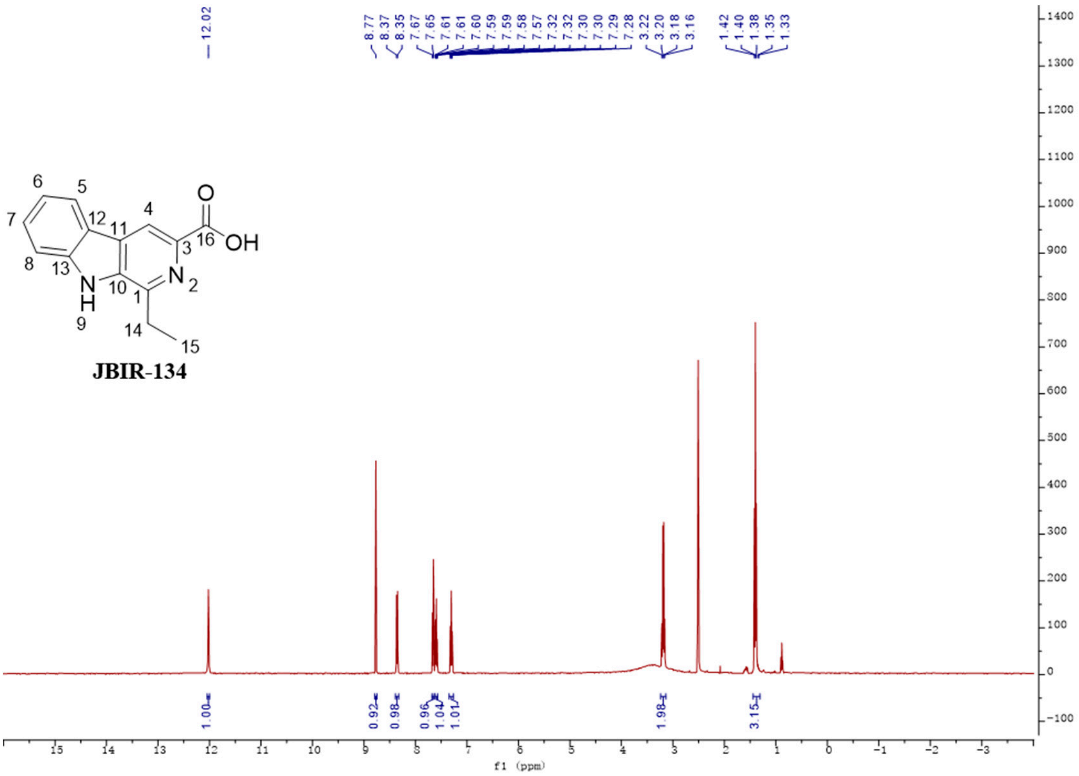

Figure S11. ^{13}C NMR (100 MHz) spectrum of JBIR-134 in DMSO.

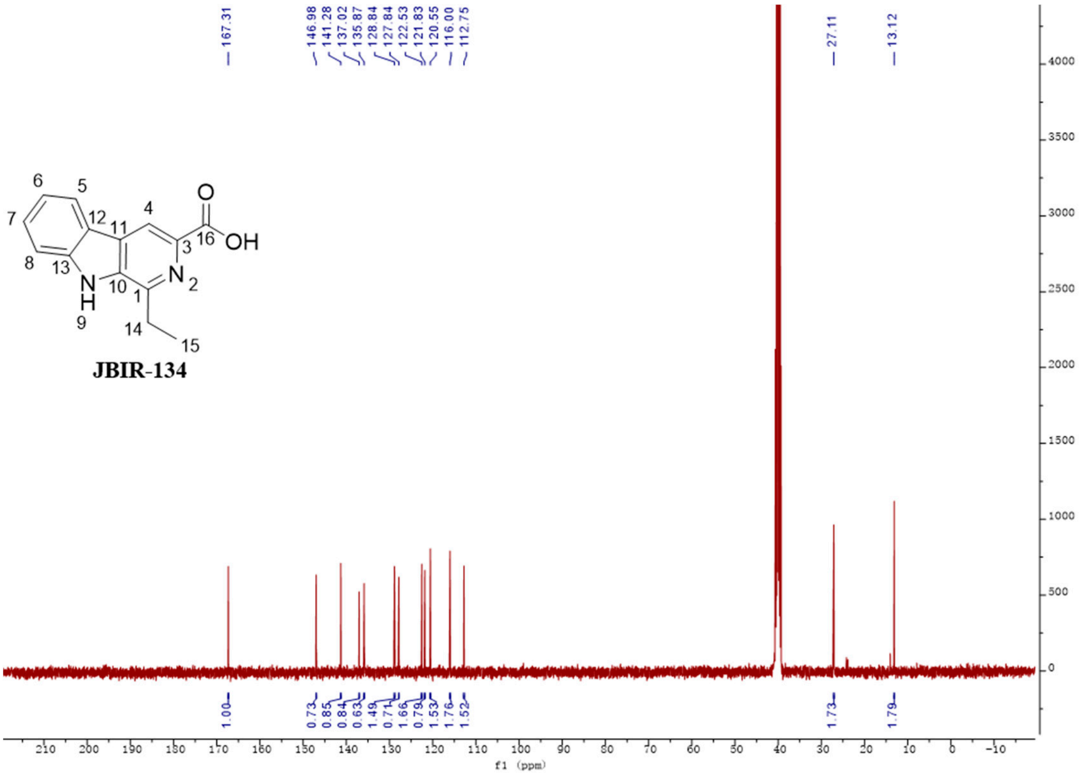

Figure S12. ESI-HRMS spectrum of kitasetaline.

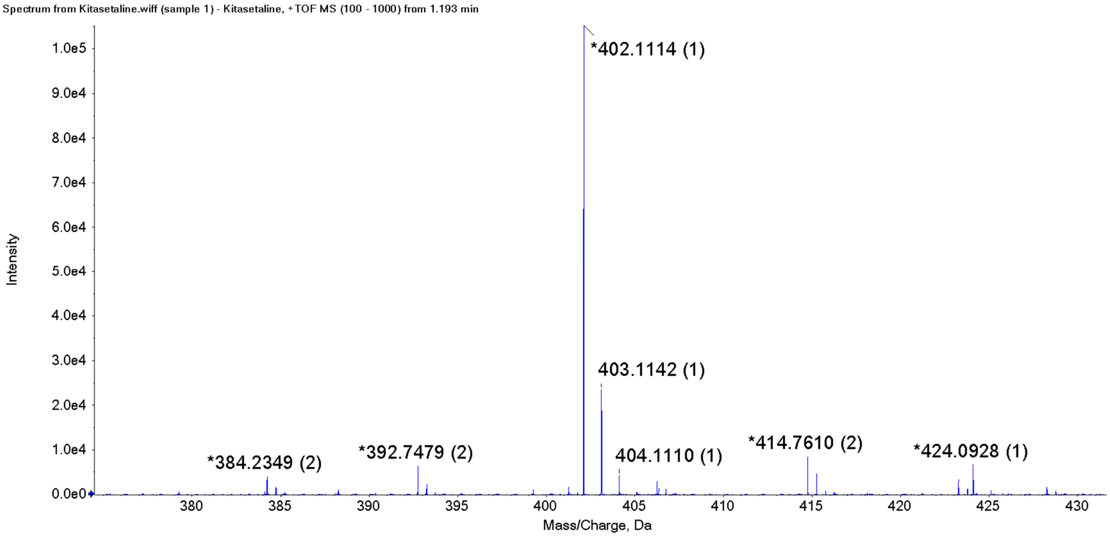

Measured *m/z*	Cal *m/z*	Error(mmu)	Ion Formula
402.1114	402.1118	-0.4	$C_{19}H_{20}N_3O_5S^+$

Figure S13. ^{1}H NMR (400 MHz) spectrum of kitasetaline in DMSO.

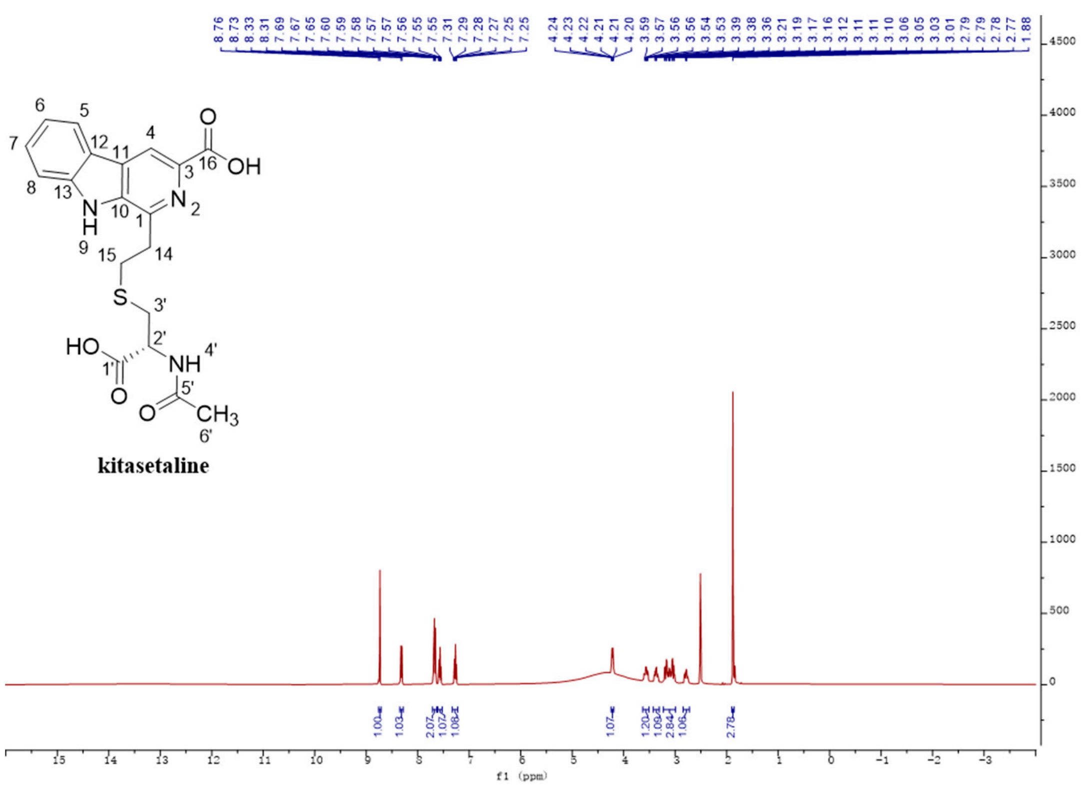

Figure S14. ^{13}C NMR (125 MHz) spectrum of kitasetaline in DMSO.

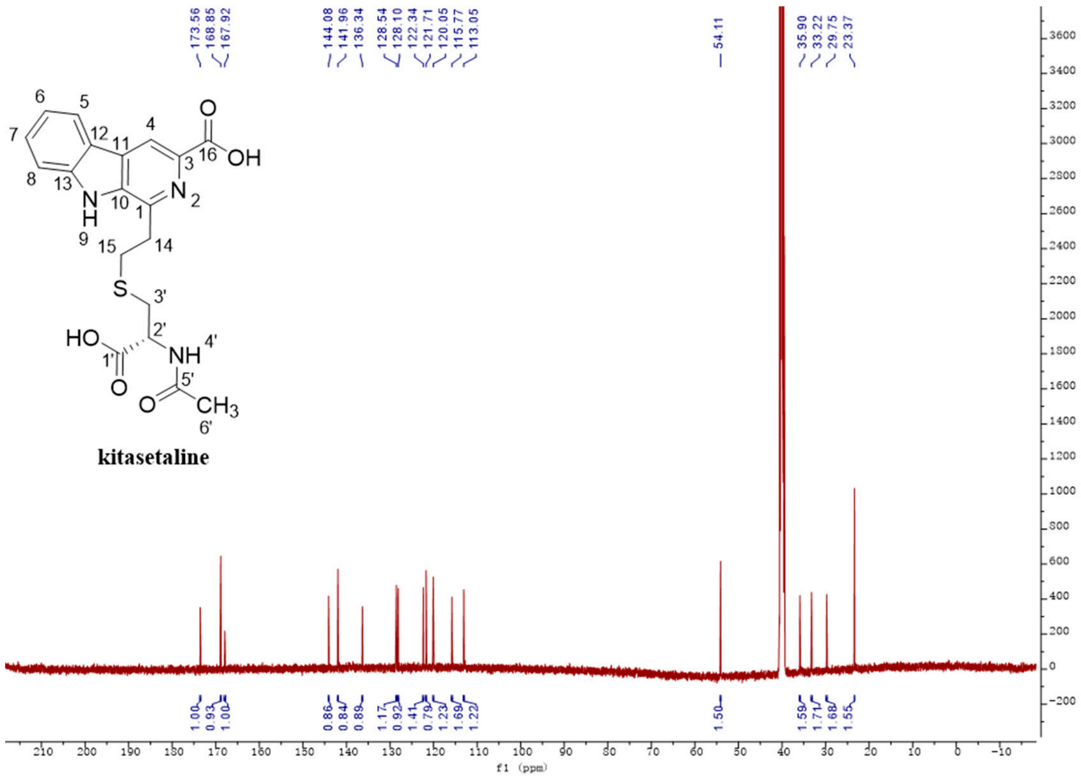

Figure S15. ESI-HRMS spectrum of novkitasetaline.

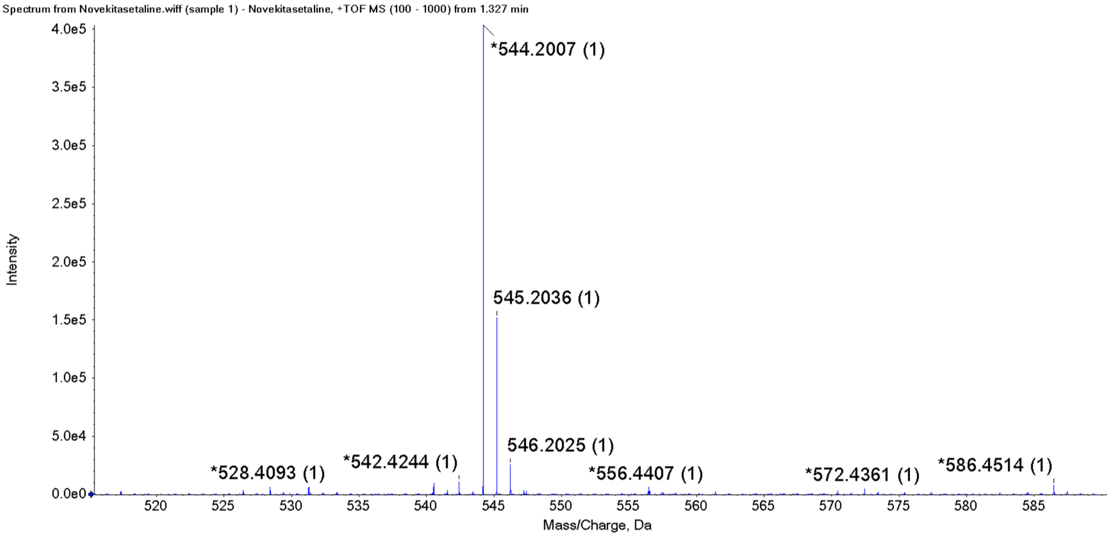

Measured *m/z*	Cal *m/z*	Error(mmu)	Ion Formula
544.2007	544.2013	-0.6	$C_{29}H_{30}N_5O_4S^+$

Figure S16. ^{1}H NMR (600 MHz) spectrum of novkitasetaline in DMSO.

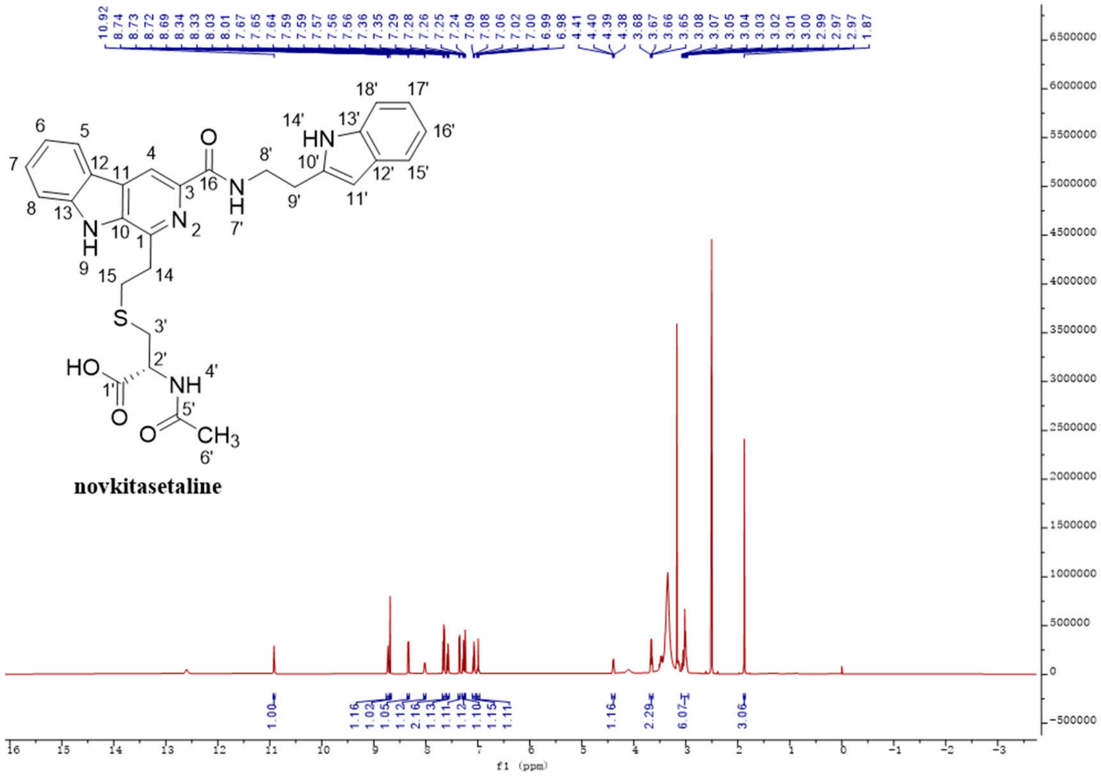

Figure S17. ^{13}C NMR (150 MHz) spectrum of novkitasetaline in DMSO.

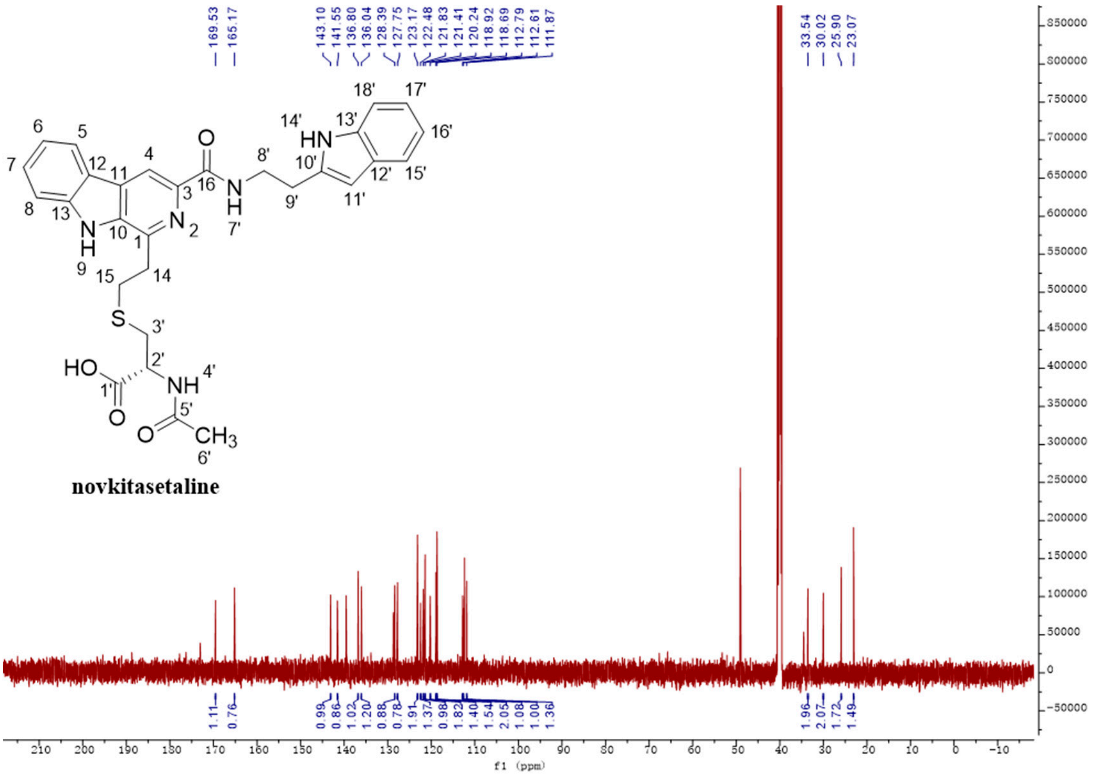

Figure S18. ^{1}H-^{1}H COSY NMR spectrum of novkitasetaline in DMSO.

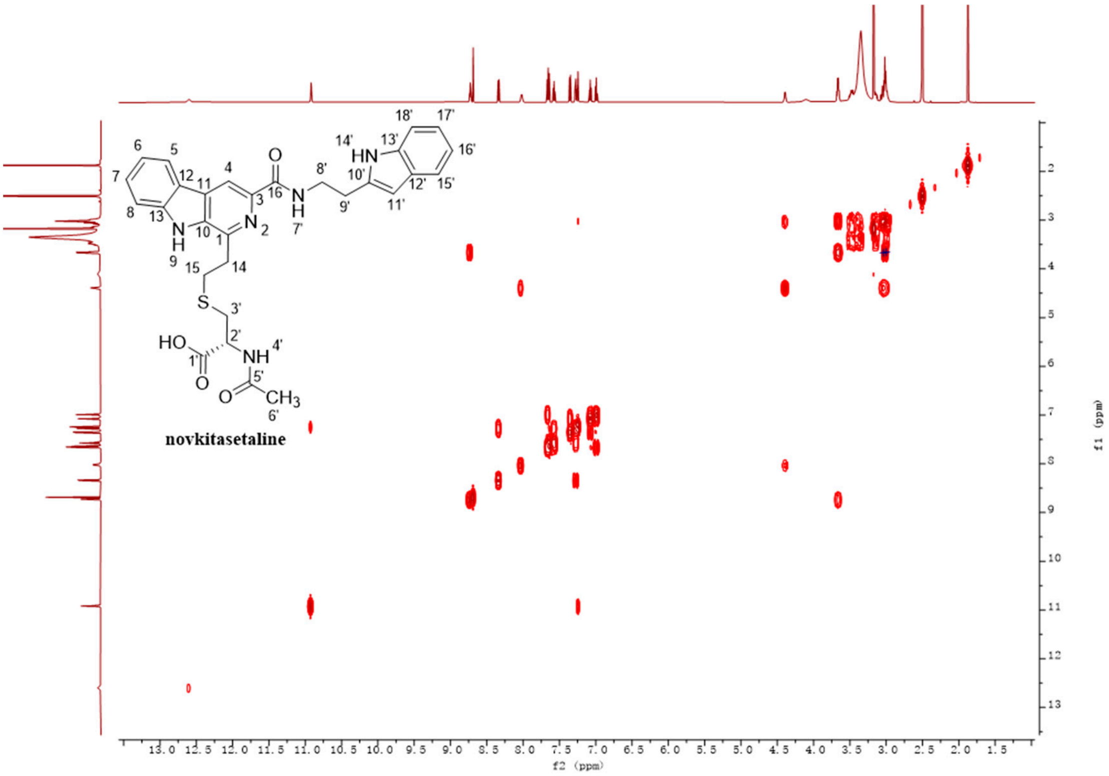

Figure S19. ^{1}H-^{13}C HMBC NMR spectrum of novkitasetaline in DMSO.

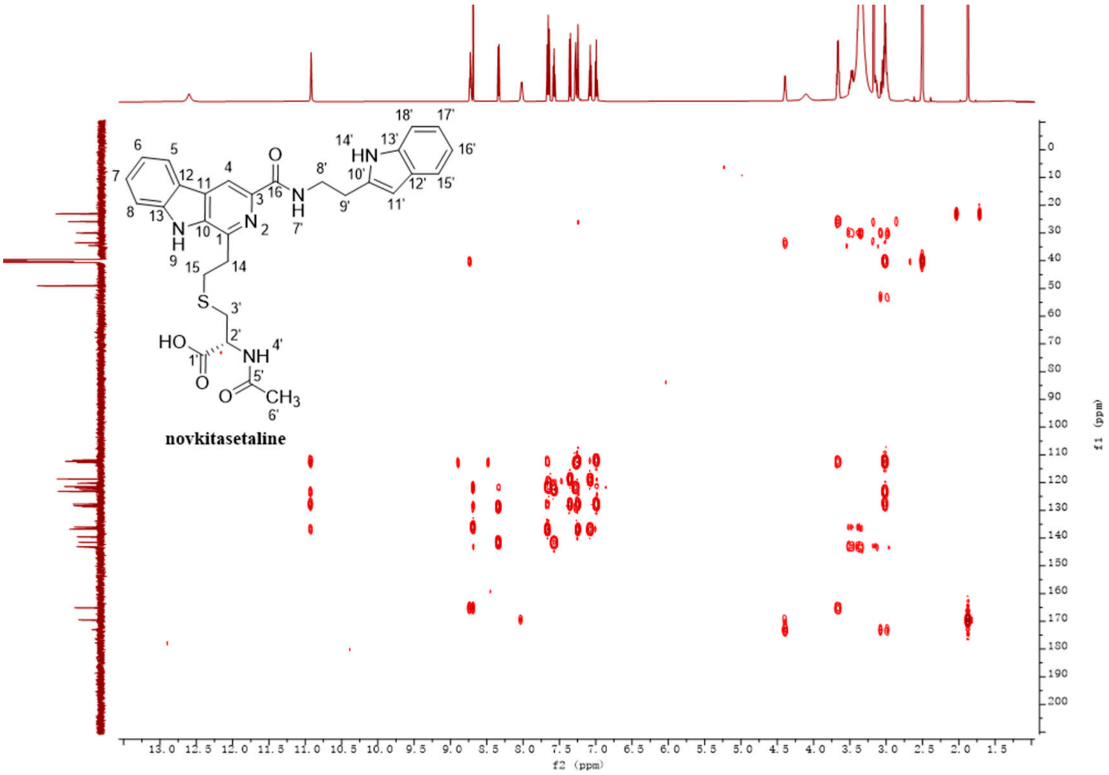

Figure S20. ^{1}H-^{13}C HSQC NMR spectrum of novkitasetaline in DMSO.

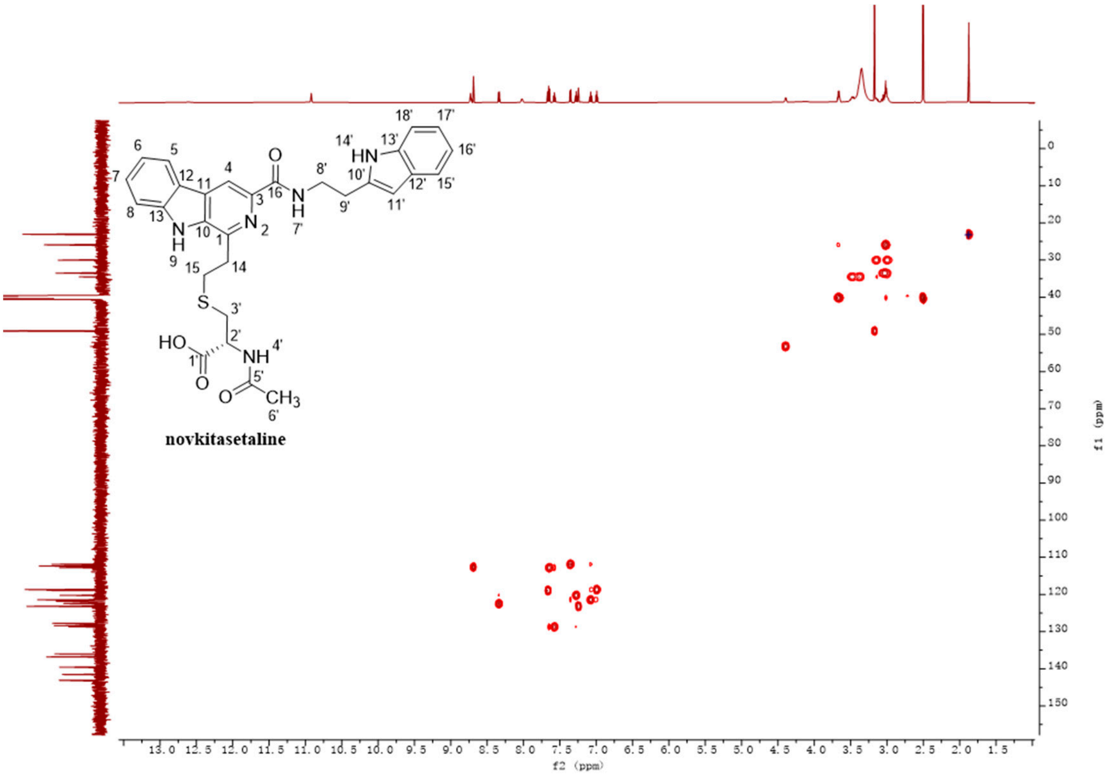

Supplement: Supplementary file 1 [file microorganisms-13-02871-s001.zip › microorganisms-3969110-supplementary.pdf]
